# Chronic Dosing with Membrane Sealant Poloxamer 188 NF Improves Respiratory Dysfunction in Dystrophic *Mdx and Mdx/Utrophin^-/-^* Mice

**DOI:** 10.1371/journal.pone.0134832

**Published:** 2015-08-06

**Authors:** Bruce E. Markham, Stace Kernodle, Jean Nemzek, John E. Wilkinson, Robert Sigler

**Affiliations:** 1 Phrixus Pharmaceuticals Inc., Ann Arbor, Michigan, United States of America; 2 Unit for Laboratory Animal Medicine, University of Michigan Medical School, Ann Arbor, Michigan, United States of America; 3 Department of Pathology and Comparative Medicine, University of Michigan Medical School, Ann Arbor, Michigan, United States of America; Ohio State University Medical Center, UNITED STATES

## Abstract

Poloxamer 188 NF (national formulary (NF) grade of P-188) improves cardiac muscle function in the *mdx* mouse and golden retriever muscular dystrophy models. However *in vivo* effects on skeletal muscle have not been reported. We postulated that P-188 NF might protect diaphragm muscle membranes from contraction-induced injury in *mdx* and mdx/utrophin^-/-^ (dko) muscular dystrophy models. In the first study 7-month old *mdx* mice were treated for 22 weeks with subcutaneous (s.c.) injections of saline or P-188 NF at 3 mg/Kg. In the second, dkos were treated with saline or P-188 NF (1 mg/Kg) for 8 weeks beginning at age 3 weeks. Prednisone was the positive control in both studies. Respiratory function was monitored using unrestrained whole body plethysmography. P-188 NF treatment affected several respiratory parameters including tidal volume/BW and minute volume/BW in mdx mice. In the more severe dko model, P-188 NF (1 mg/Kg) significantly slowed the decline in multiple respiratory parameters compared with saline-treated dko mice. Prednisone’s effects were similar to those seen with P-188 NF. Diaphragms from P-188 NF or prednisone treated *mdx* and dko mice showed signs of muscle fiber protection including less centralized nuclei, less variation in fiber size, greater fiber density, and exhibited a decreased amount of collagen deposition. P-188 NF at 3 mg/Kg s.c. also improved parameters of systolic and diastolic function in *mdx* mouse hearts. These results suggest that P-188 NF may be useful in treating respiratory and cardiac dysfunction, the leading causes of death in Duchenne muscular dystrophy patients.

## Introduction

Duchenne muscular dystrophy (DMD) is a genetic disorder that occurs with a frequency of approximately 1 in every 3500 live male births [1] resulting from mutations in the dystrophin gene, located on the short arm of the X chromosome [2–4]. Affected boys are usually diagnosed at 3–5 years of age with symptoms of delayed walking or gait disturbances progressing to general muscle weakness and eventually death [5]. Genetic testing for mutations in the gene encoding dystrophin is used to confirm the diagnosis. In the later stages of the disease, a network of fibrous connective tissue and adipose tissue replaces muscle fiber lost due to necrosis, leaving only small islands of intact muscle fibers. Over time the muscles become progressively weaker, and the use of a wheelchair becomes necessary at a mean age of 9.5 years [5, 6]. Weakness in the diaphragm and intercostal muscles impairs respiratory function, which is the major cause of death in this patient population. The second most frequent cause of death is heart failure due to dilated cardiomyopathy. [7–11].

Dystrophin has been shown to protect the sarcolemma from contraction-induced damage. Evidence for this comes from the *mdx* mouse model, where muscle cells show increased permeability, increased intracellular calcium, and increased susceptibility to osmotic shock when stretched [12–14]. In cardiomyocytes from *mdx* mice, the membrane sealant P-188 NF has been shown to interact with exposed hydrophobic regions of fragile membranes and prevent unregulated entry of extracellular Ca^2+^ into the muscle cell, thereby restoring normal tension development in *mdx* cardiomyocytes [14]. The exact nature of the exposed hydrophobic regions was not identified but could occur from either direct microscopic tears in the lipid portion of the membrane [14] or from alterations in membrane protein composition, or activity, that has been shown to occur in this dystrophic model [15–19]. In any case, the resulting dysregulation of calcium in heart muscle resulted in abnormal heart structure and function [14]. The authors suggested that P-188 NF acts as a molecular “Band-aid” to seal the tears and permit intracellular calcium levels to return to normal [14]. The ability of P-188 NF to improve intracellular calcium concentration also translated into improved heart structure and function in the GRMD dog model of muscular dystrophy [20].

While P-188 NF has been shown to improve cardiac muscle function, its effects on skeletal muscle have been more difficult to demonstrate with some apparently contradictory results. P-188 NF did not prevent uptake of Evan’s Blue dye in rectus femoris muscle in exercised *mdx* mice [21] but did prevent uptake in Tibialis Anterior (TA) muscle [22]. While it was effective in preventing force decline during isometric contractions of isolated *mdx* mouse lumbrical muscle [23], it was not found to be effective, *in situ*, in Tibialis Anterior muscle, when administered intraperitoneally (i.p.) and in combination with anesthesia [24], although P-188 NF did protect muscle cells in the latter model. Further it did not reduce plasma creatine kinase levels in the GRMD dog model of muscular dystrophy [20] when dosed chronically. Regardless of whether or not P-188 NF can protect dystrophic limb muscles from contraction-induced damage, diaphragm muscle is arguably the most important skeletal muscle to protect in muscular dystrophy because respiratory failure is the leading cause of death for patients with DMD (6). The diaphragm, which is one of the first skeletal muscles to deteriorate in *mdx* mice [25] is also highly vascularized [26] which should permit high exposure to P-188 NF.

The studies reported here were undertaken to determine if P-188 NF could protect diaphragm muscle and prevent further respiratory dysfunction. The effect of P-188 NF on respiratory deficits in unanesthetized *mdx* and *mdx*/utrophin^-/-^ (dko) mice was monitored *in vivo* using unrestrained whole body plethysmography (WBP). The treatment regimen for the *mdx* mice was initiated at 7 months ± 2 weeks of age, a time when respiratory deficits and diaphragm damage are already present in the *mdx* mouse model [27–32], and continued for 22 weeks until the mice were 1 year of age. The dosing regimen used in the dkos was initiated at 3 weeks ± 3 days of age and continued for 8 weeks. Subsequently, diaphragm muscles were evaluated histologically. We show here that P-188 NF treatment had an impact on several respiratory parameters in the *mdx* and dko mice with respect to baseline and/or the saline-treated control group. Further, P-188 NF treatment significantly reduced the number of centralized nuclei, variance in the minimal Feret’s diameter [33, 34], and collagen deposition suggesting that P-188 NF slows the degeneration process in dystrophic diaphragm muscle. These results indicate that P-188 NF might delay the loss of respiratory activity in DMD patients.

## Materials and Methods

### Animals

Male C57BL/10SnJ, hereafter referred to as wild type, and male *mdx* C57BL/10ScSn-Dmd^*mdx*^ mice, were purchased from Jackson Laboratory, and aged in University of Michigan (UM) facilities. Heterozygous *mdx*/utrophin^+/-^ mice were purchased from Jackson Laboratory (STOCK Utrn^*tm1Jrs*^ Dmd^*mdx*^/J; stock number 016622) and bred at the breeding colony facility at the UM. All studies were performed in facilities run by the UM Unit for Laboratory Animal Medicine (ULAM). Mice were housed on a 12 hr dark-light cycle and provided water and chow ad libitum. All ages for *mdx* mice are ± 2 weeks and dko mice are ± 3 days.

All animal work was approved by the University of Michigan University Committee on the Use and Care of Animals (PRO00005303) and all studies were performed in facilities run by the UM ULAM. The facility holds an Office of Laboratory Animal Welfare-approved assurance from the NIH. All studies conformed to the Guide for the Care and Use of Laboratory Animals by the National research council and the International Guiding Principles for Biomedical Research Involving Animals by the International Council for Laboratory Animal Sciences. Euthanasia was performed as recommended by the AVMA Guidelines for the Euthanasia of Animals. Finally 3 of the 5 authors hold DVM degrees. Isoflurane was used as an anesthetic for performance of echocardiography. No other studies with the animals required anesthesia.

Dko mice were housed in cages with a heat strip running across the bottom of the cage which was adjusted such that the temperature at the front of the cage was 37° ± 3° while the back of the cage was at room temperature. These mice were fed DietGel (Clear H_2_O; Portland, ME) in containers placed on the cage bottom, in addition to access to normal chow, and provided with nesting material (Envirodry). In spite of these extra measures, attrition was high in the dko groups.

At 7 months of age for *mdx* mice, treatment with P-188 NF, saline or prednisone was initiated. All compounds were administered subcutaneously (s.c.). Animals were dosed once daily (QD) and received saline, P-188 NF at 3 mg/Kg or prednisone at 1 mg/Kg in a volume of 0.1 ml. For dko mice, P-188 NF (1 mg/Kg), prednisone (1 mg/Kg) or saline were administered QD, s.c. in a volume of 0.1 ml.

### Respiratory measurements

Respiration was monitored using a Buxco Whole Body Plethysmography apparatus (Buxco, Troy, NY) according to the manufacturers instructions as modified by the WBP protocol from Treat-NMD [35]. In this protocol animals are un-restrained and conscious. Monitoring was performed in the room in which the animals were housed. The *mdx* mice were monitored for 2 months prior to initiation of treatment to acclimate the mice to the procedure. Mice were placed in monitoring chambers and allowed to acclimate for 15 minutes, until they were quiet and motionless, prior to recording respiratory parameters. Longer acclimation times up to 45 minutes did not change the respiratory results. Each mouse was monitored in the same chamber for each reading throughout the study to minimize variability. During acclimation and monitoring the room door was shut, and technician movement and room noise was kept to a minimum. All readings were performed between the hours of 7 and 11 AM. After the acclimation period, respiratory function was monitored for 15 minutes. Mice were assigned to groups based on tidal volume (TV) measurements made the week before taking baseline respiratory measurement. TV was chosen to normalize the groups because at the time it was one parameter that was expected to change at the time the studies were initiated [29]. TV value averaging was accomplished by randomly placing 16 *mdx* mice into each group and removing mice with high or low TV values until the mean TV values per group were not significantly different. This resulted in a final group size of 12. Upon initiation of dosing, mice were monitored every 2 weeks.

### Echocardiography

Induction of anesthesia was performed on the 1-year-old mice in an enclosed container containing 5% isoflurane. After induction, the mice were placed on a warming pad to maintain body temperature. 1–1.5% isoflurane was supplied via a nose cone to maintain a surgical plane of anesthesia. The hair was removed from the upper abdominal and thoracic area with depilatory cream. ECG was monitored via non-invasive resting ECG electrodes. Transthoracic echocardiography was performed in the supine or left lateral position. Two-dimensional, M-mode, Doppler and tissue Doppler echocardiographic images were recorded using a Visual Sonics’ Vevo 2100 high resolution in vivo micro-imaging system. LV ejection fraction was measured from the two-dimensional long axis view. In addition systolic and diastolic dimensions and wall thickness was measured by M-mode in the parasternal short axis view at the level of the papillary muscles. Fractional shortening and ejection fraction were also calculated from the M-mode parasternal short axis view. Diastolic function was assessed by conventional pulsed-wave spectral Doppler analysis of mitral valve inflow patterns (early [E] and late [A] filling waves). Doppler tissue imaging (DTI) was used to measure the early (Ea) diastolic tissue velocities of the septal annulus and lateral annulus of the mitral valve in the apical 4-chamber view.

### Data analysis and Statistics

Data were monitored and collected using FinePoint software purchased from Buxco Electronics. The data were transferred to GraphPad Prizm software for graphing and statistical analysis of the data. Comparisons between groups were done by two-way (treatment and time), repeated measures ANOVA except for baseline data, which were analyzed by one-way ANOVA using a Tukey post-hoc test. Significance was set at P < 0.05. For *mdx* mice, N = 12 animals per group except for the *mdx* 3 mg/Kg group which had only 11 animals on weeks 20 and 22. For the dko mice, the starting group sizes varied depending upon the supply of mice in the colony but the maximum number of mice available (≥ 20) was used in order to maximize the number of survivors at the end of the study. For the dko study where mortality was approximately 80% during the 8-week dosing schedule, only the respiratory data from the surviving mice are represented with the exception of dko baseline data in the S1 Table.

Abbreviations used include the following (units): Measured parameters: F, respiration rate (breaths/minute), TV, tidal volume, (ml); MV, minute volume, (ml/min); Penh, enhanced pause, (no units); PIF, peak inspiratory flow, (ml/sec); PEF, peak expiratory flow, (ml/sec); Te, expiratory time (sec); Ti, inspiratory time, (sec); Tr, relaxation time, (sec); and derived: TV/BWT, ratio of TV to body weight, (ml/Kg); Rpef, the ratio of the time from start of expiration to peak expiratory flow to Te (no units).

### Histology

At the end of treatment, diaphragm muscles were removed from the mice, rinsed with ice cold PBS, formalin fixed, and embedded in paraffin. Sections (4 μm) were prepared and stained by the Histology Core at the UM. Staining procedures for the diaphragm included H&E, picosirius red, wheat germ agglutinin (WGA), and DAPI. Overlapping microscopic (10X) images (4–5 per section) were prepared for evaluation. Diaphragm muscle was cross-sectioned from regions near each end and near the middle to evaluate structure throughout the muscle. Longitudinal sections were also prepared. A minimum of 10K fibers per *mdx* mouse (n = 11 or 12) was analyzed. For dko mice, a minimum of 4 diaphragm muscles were analyzed per group with 5 sections per muscle. This resulted in a minimum of 1500 fibers being analyzed per group. Pathologists, blinded to sample identity evaluated the slides for centralized nuclei (CN), fiber density, fibrosis and (VC) coefficient of the minimal Feret’s diameter, a surrogate for cross sectional area [33, 34]. WGA-DAPI stained sections were analyzed using fluorescent images and Nikon Elements software (computer-assisted determination of fiber area) to evaluate the minimum Feret’s diameter from each fiber. The variance coefficient of the minimum Feret’s diameter was used as measure of improvement in muscle fiber size to avoid sectioning artifacts as recommended by the Treat-NMD neuromuscular network and others [33, 34].

### Pharmacokinetic studies

C56BL/6 mice received either a single intravenous (i.v.) or an s.c. dose of P-188 NF an i.v. dose levels of 4.6 mg/kg and s.c. dose levels of 3.0 mg/kg. C56BL/6 mice received either a single intravenous or an s.c. dose of P-188 NF at intravenous dose levels of 4.6 mg/kg and s.c. dose levels of 3.0 mg/kg. Blood was collected from a group of ten animals per time-point at 0.083, 0.25, 0.5, 1, 2, 4, 8 and 12 h after dosing for the preparation of plasma from which the P-188 NF concentrations were determined by liquid chromatography-mass spectrometer methods developed at Millennium Research Laboratories, Inc. (Woburn, MA)

The plasma profiles were constructed from the mean of the plasma concentrations for each sample collection time, dose level and dose route. For the purposes of the pharmacokinetic analysis, all samples at <0 in the bioanalytical report were set to zero. All other non-numerical data were excluded from further analysis. The pharmacokinetics of the plasma profiles of the individual animals for each dose route and dose level were analyzed using a non-compartmental approach for an intravenous or extravascular dose, as appropriate, with WinNonlin ‘v’ 5.2 (Pharsight, CA). Cmax and Tmax were taken directly from the data and the AUC_last_ calculated using the linear trapezoidal rule for the ascending portion of the plasma profile and the log trapezoidal rule for the descending portion. The half-life was determined from a linear regression of the logarithm of the P-188 NF plasma concentration versus time. The number of points used for the regression was determined from visual inspection of the data and used a minimum of 3 time points. The data were collated and tabulated, together with descriptive statistics, using WinNonlin (Certera, Princeton, NJ).

## Results

### Selection of dosage and route of administration

P-188 NF has been shown to have cell protective effects in several models of cell damage and in animal disease models [14, 36–39]. However, most of the *in vivo* work was performed using relatively high doses, 200–460 mg/Kg, and administered by i.v. or i.p. routes of administration [14, 20, 21, 40, 41]. In order for P-188 NF to become a viable therapy it must be demonstrated to work at a dosage and by a route of administration that is acceptable for daily administration to patients. Previous work in the coronary microembolism model of heart failure has shown that P-188 NF is active at an i.v. dose of 15 mg/Kg [42]. In addition, P-188 NF was shown to prevent muscle atrophy in the dysferlin-deficient SJL mouse model of limb-girdle muscular dystrophy using the s.c. route of delivery at an effective daily dose of approximately 16 mg/Kg [43]. In addition, Phrixus has data demonstrating acutely improved cardiac hemodynamics in a rat heart failure model at 4.6 mg/Kg (S1 Fig).

A pharmacokinetic (PK) study was performed with non-labeled P-188 NF to compare the PK of P-188 NF via the i.v. and s.c. routes of administration (Table 1). The doses studied were 4.6 mg/Kg i.v. (active in rat) and 3 mg/Kg s.c. It can be seen in Table 1 that the plasma exposure to P-188 NF (area under the curve (AUC) was greater in the 3 mg/Kg s.c. dose than with the 4.6 mg/Kg i.v. dose. The greater exposure at 3 mg/Kg s.c. suggests that this dose might have activity in mouse models.

**Table 1:** Pharmacokinetic parameters of P-188 NF dosed by the intravenous or subcutaneous routes of administration in mice.

Parameters*	Dose level (mg/kg)	t_1/2_ (h)	C_max_ (μg/mL)	T_max_ (h)	AUC_last_ (μg*h/mL)	AUC_∞_ (μg*h/mL)	F (%)
**Intravenous**	**4.6**	8.2	23.0^1^	NC	20.3	25.9	NC
**Subcutaneous**	**3.0**	3.9	7.0	2	36.9	42.3	94.1

* Cmax, maximum plasma concentration; Tmax, time to maximum plasma concentration; AUClast, area under the curve up to the last measurement; AUC∞, area under the curve extrapolated to infinity; F, bioavailbility. NC-not calculated.

^1^ Calculated based on a 30g mouse with 6 ml of blood.

### Respiratory function is diminished in *mdx* mice at 7 months of age

Baseline respiratory values (Table 2) were taken at the end of an acclimation period when the mice were 7 months of age ± 2 weeks. The mice were grouped based on baseline TV values since this value was expected to decline over time based on a literature report [29]. At 7 months of age there were several significant differences in respiratory function between wild type and *mdx* groups at baseline, which were similar to differences reported previously for *mdx* mice at 6 and 7 months of age [28, 29] including, TV, MV, PEF and Rpef, a parameter influenced by both airway obstruction and residual activity of inspiratory muscles during the initiation of expiration. Body weights of the *mdx* mice tended to be larger than those of the wild type mice with the 3 mg/Kg P-188 NF group being significantly larger (Table 2).

**Table 2:** Comparison of baseline respiratory function and body weight between groups of wild type and *mdx* mice at age 7 months^g^.

Parameter	Wild type Saline	*mdx* Saline	*mdx* P-188 NF 3 mg/Kg	*mdx* Prednisone
F (breaths/min)	426 ± 27^d^	391 ± 19^a^	414 ± 28	404 ± 16
TV (ml)	0.324 ± 0.024	0.314 ± 0.017	0.323 ± 0.021	0.321 ± 0.019
TV/BW (mg/g)	0.0093± 0.0008	0.0084 ± 0.0005	0.0083 ± 0.0005^a^	0.0088± 0.0005
MV (ml/min)	137 ± 10^d^	121 ± 10^a^	132 ± 11	127 ± 8
MV/BW (ml/min/g)	4.01 ± 0.32^f^	3.41 ± 0.20^c^	3.59 ± 0.24^c^	3.61 ± 0.18^b^
Penh	0.427 ± 0.084	0.424 ± 0.043	0.443 ± 0.057	0.470 ± 0.046
Rpef	0.404 ± 0.049^d^	0.325 ±0.048^b^	0.350 ± 0.060^a^	0.335 ± 0.027^b^
PIF (ml/sec)	10.89 ± 0.82	10.18 ± 0.72	10.70 ± 0.73	10.27 ± 0.63
PEF (ml/sec)	5.36 ± 0.49^d^	4.80 ± 0.40^a^	5.23 ± 0.46	5.10 ± 0.42
Ti (sec)	0.049 ± 0.003	0.052 ± 0.002	0.051 ± 0.003	0.053 ± 0.002^a^
Te (sec)	0.114 ± 0.014	0.128 ± 0.011	0.118 ± 0.013	0.123 ± 0.009
BW (g)	33.0 ± 2.5	35.4 ± 1.9	36.9 ± 2.3^c^	35.2 ± 2.3

^a^ P < 0.05 vs. wild type saline.

^b^ P = 0.001–0.01 vs. wild type saline.

^c^ P < 0.001 vs. wild type saline.

^d^ P < 0.05 vs. *mdx* saline.

^e^ P = 0.001–0.01 vs. *mdx* saline.

^f^ < 0.001 vs. *mdx* saline.

^g^ all values are mean ± S.D. Analysis by 2-way ANOVA using a multiple comparison (simple effects within rows) post-hoc test.

### Chronic treatment with Poloxamer 188 NF improves respiratory function in *mdx* mice with established respiratory dysfunction

**Fig 1:**
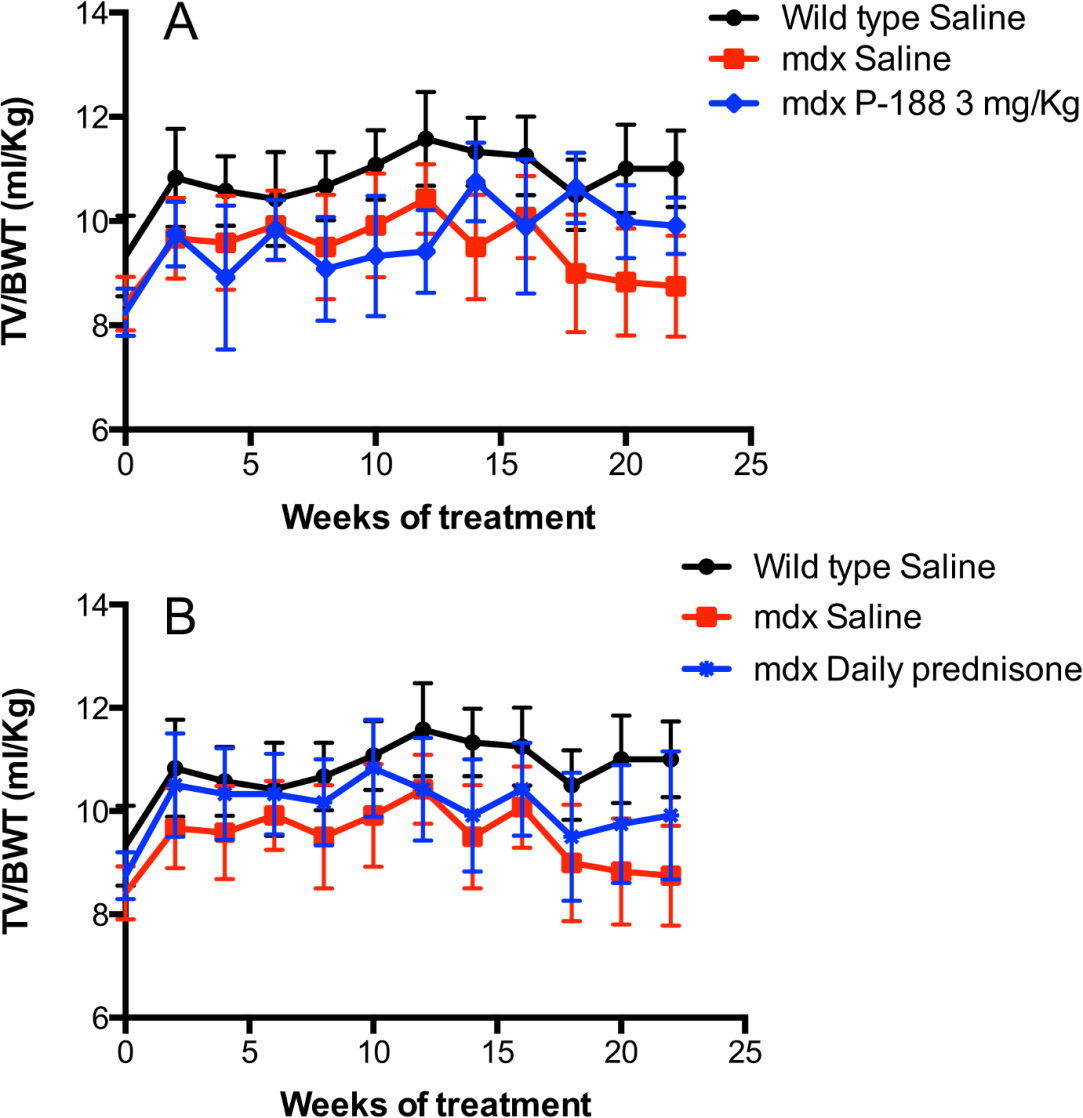
Effects of P-188 NF and prednisone on tidal volume/BW in the *mdx* mouse over time. *Mdx* mice were treated QD, *s*.*c*. with P-188 NF (3 mg/Kg) or prednisone (1 mg/Kg) from age 7 months to 12 months ± 2 weeks. The black line represents wild-type mice (C57BL/10 SnJ) treated with saline. The red line represents *mdx* mice treated with saline. The blue line represents mice treated with P-188 NF (Panel A) or 1 mg/Kg prednisone (Panel B). Data points are means +/- S.D. N = 11 for *mdx* 3 mg/Kg P-188 NF at 20 and 22 weeks, N = 12/group for prednisone treatment. Panel A, P < 0.0001 for the overall effect of P-188 NF 3 mg/Kg group vs. *mdx* saline and wild type saline. The *mdx* 3 mg/Kg group was also significantly greater than *mdx* saline at 22 weeks (P < 0.0001). Panel B, P = 0.001 to 0.0001 for *mdx* 1 mg/Kg prednisone group vs. *mdx* saline and P < 0.0001 vs. wild type saline. The *mdx* prednisone group was also significantly greater than *mdx* saline at 22 weeks (P < 0.0001).

Wild type mice were dosed once per day s.c with saline while *mdx* mice were dosed once per day s.c. with saline, 3 mg/kg of P-188 NF or 1 mg/kg of prednisone for 22 weeks. Respiration was monitored every 2 weeks by WBP. Fig 1, shows the overall effect of P-188 NF and prednisone treatment on TV/BW ratio, a parameter dependent upon TV, which was used to assign animals to groups. The TV/BW ratios were nearly identical at the start of the study (Table 1) and increased in both the saline and P-188 NF treated groups over the first 12 to 14 weeks of the study. The reason for this improvement is not known but could be related to the increase in fluid that both groups received from dosing. During that time (from 4–12 weeks), it appears that the saline treated group had higher TV/BW ratios, however, the saline-treated *mdx* mice had significantly higher TV/BW ratios (P < 0.05) only at the 12-week time point. Subsequently the 3 mg/Kg doses of P-188 NF prevented a nearly 20% decline seen in the saline-treated group that occurred from 12 to 22 weeks. It is also noteworthy that TV/BW ratio was significantly increased after 22 weeks of treatment with P-188 NF (P = 0.001 to 0.0001) or prednisone (P < 0.05), but not saline, compared to baseline. In the prednisone-treated group of *mdx* mice, treatment increased TV/BW early in the dosing regimen, which was maintained except at week 14 where the TV/BW was similar between the saline and prednisone-treated *mdx* groups. This slowing of the decline was also seen with MV/BW ratio (S11 Fig), however neither P-188 NF nor prednisone treatment improved MV/BW over baseline. Table 3 shows the overall statistically significant effects of 22 weeks of treatment on respiratory parameters. Highly statistically significant differences between the wild type saline and *mdx* saline groups were observed, over time, in all parameters with the exception of expiration time (Te). P-188 NF had significant effects on TV, TV/BW, MV, MV/BW, and PIF compared to saline treated *mdx* mice (S1–S11 Figs). Prednisone treatment, which delays the need for respiratory assistance in DMD patients [5, 6], had significant effects on TV, TV/BW, MV, MV/BW, Penh, Rpef, PIF, Ti, Te, and Tr in this model. For both P-188 NF and prednisone treatments the significance of the overall effect was established over time.

**Table 3:** The main treatment effect on respiratory function in *mdx* mice over 22 weeks of treatment with saline or P-188 NF.

	Respiratory Function										
Two-way ANOVA^1^ ^2^	F	TV	TV/BW	MV	MV/BW	Penh	Rpef	PIF	PEF	Ti	Te
Wild type saline	****	****	****	****	****	****	****	****	****	****	ns
*mdx* saline	nd	nd	nd	Nd	nd	nd	nd	nd	nd	nd	nd
*mdx* 3 mg/Kg P-188 NF	*	**	**	**	*	**	**	****	ns	**	ns
*mdx* prednisone	ns	**	**	*	***	****	****	****	ns	**	*

^1^ Two-way ANOVA using a multiple comparisons (main treatment effect) post-hoc test.

^2^ All comparisons to *mdx* saline group; nd, not different; ns not significant.

**** P < 0.0001.

*** P = 0.001 to 0.0001.

** P = 0.01 to 0.001.

* P < 0.05.

### P-188 NF improves cardiac hemodynamics in the *mdx* mouse model when administered subcutaneously.

Since P-188 NF has been previously shown to improve heart function when administered i.v. [14, 20, 40–42], it was of interest to determine if the same doses and route of administration that affected respiratory function in the *mdx* mouse would improve heart function in the same model. Therefore, the mice used in the respiratory study were monitored by echocardiography at the end of the 22-week treatment period. The results are shown in Table 4. Compared with the saline-treated *mdx* mouse group, P-188 NF treatment caused a significant increase in fractional shortening (FS), ejection fraction (EF), stroke volume (SV), and the related cardiac output (CO). In addition, treatment with P-188 NF caused a significant decreased isovolumic relaxation time. Based on a comparison with saline treatment, P-188 NF treatment at this dose and route of administration did not affect heart rate (HR) or measures of volume or wall thickness (Table 4). The results indicate that P-188 NF delivered QD s.c. at a dose of 3 mg/Kg was sufficient and appropriate to affect an improvement in heart function.

**Table 4:** Echocardiography results from wild type and *mdx* mice treated with saline or P-188 NF for 22 weeks from 7 months to 1 year of age.

Parameters**	Wild type saline	*mdx* saline	*mdx* 3 mg/Kg	*mdx* prednisone
BW (g)	33 ± 4	33 ± 3	34 ± 2	32 ± 2
HR (b/m)	406 ± 48	370 ± 51	410 ± 38	376 ± 50
E/A	1.8 ± 0.5	1.6 ± 0.2	1.5 ± 0.2	1.7 ± 0.9
IVRT (ms)	21 ± 3	23 ± 3	18 ± 2*	21 ± 3
EF (%)	49 ± 5	50 ± 4	58 ± 9*	56 ± 7
FS (%)	25 ± 3	24 ± 3	31 ± 6*	26 ± 4
LVDd (μl)	4.5 ± 0.2	4.1 ± 0.3	4.1 ± 0.3	4.0 ± 0.2
LVvol d (μl)	91 ± 10	78 ± 13	73 ± 7	70 ± 8
IVSd (mm)	0.8 ± 0.09	0.8 ± 0.07	0.09 ± 0.07	0.09 ± 0.07
PWd (mm)	0.7 ± 0.1	0.9 ± 0.06	0.9 ± 0.07	0.8 ± 0.09
SV (μl)	37 ± 8	34 ± 5	44 ± 6*	35 ± 8
CO (ml/m)	19 ± 4	15 ± 4	21 ± 4*	17 ± 6

* P < 0.05 vs. mdx saline.

** All mice were 1 year old with 22 weeks treatment.

While the *mdx* mouse is an accepted model of muscular dystrophy and considered to be an appropriate model for drug testing by Treat-NMD, the model is limited by the fact that it has a mild phenotype [44]. In order to examine the effects of P-188 NF in a more severe mouse model, we obtained the heterozygous *mdx*/utrn^+/-^ (het) mice, and mated the het mice to obtain *mdx*/utrn^-/-^ (dko) mice which exhibit a much more severe muscular dystrophy phenotype.

### P-188 NF treatment significantly slows the decline in respiratory parameters in dko mice

Dko mice are fragile and have a severely truncated life span [45–47] so in order to have enough mice survive to the end of the study groups were populated with 20 or more mice. Because of this, we performed dko studies with only a single treatment at a time and included wild type mice and dko mice treated with saline as controls with each treatment. P-188 NF treatment was run twice in order to determine reproducibility of the effects. The data were combined from these studies for analysis. Finally, while performing some pilot studies with these mice, we observed respiratory effects of P-188 NF dose of 1 mg/Kg. Based on this observation, 1 mg/Kg was chosen as the dose for the dko studies.

**Fig 2:**
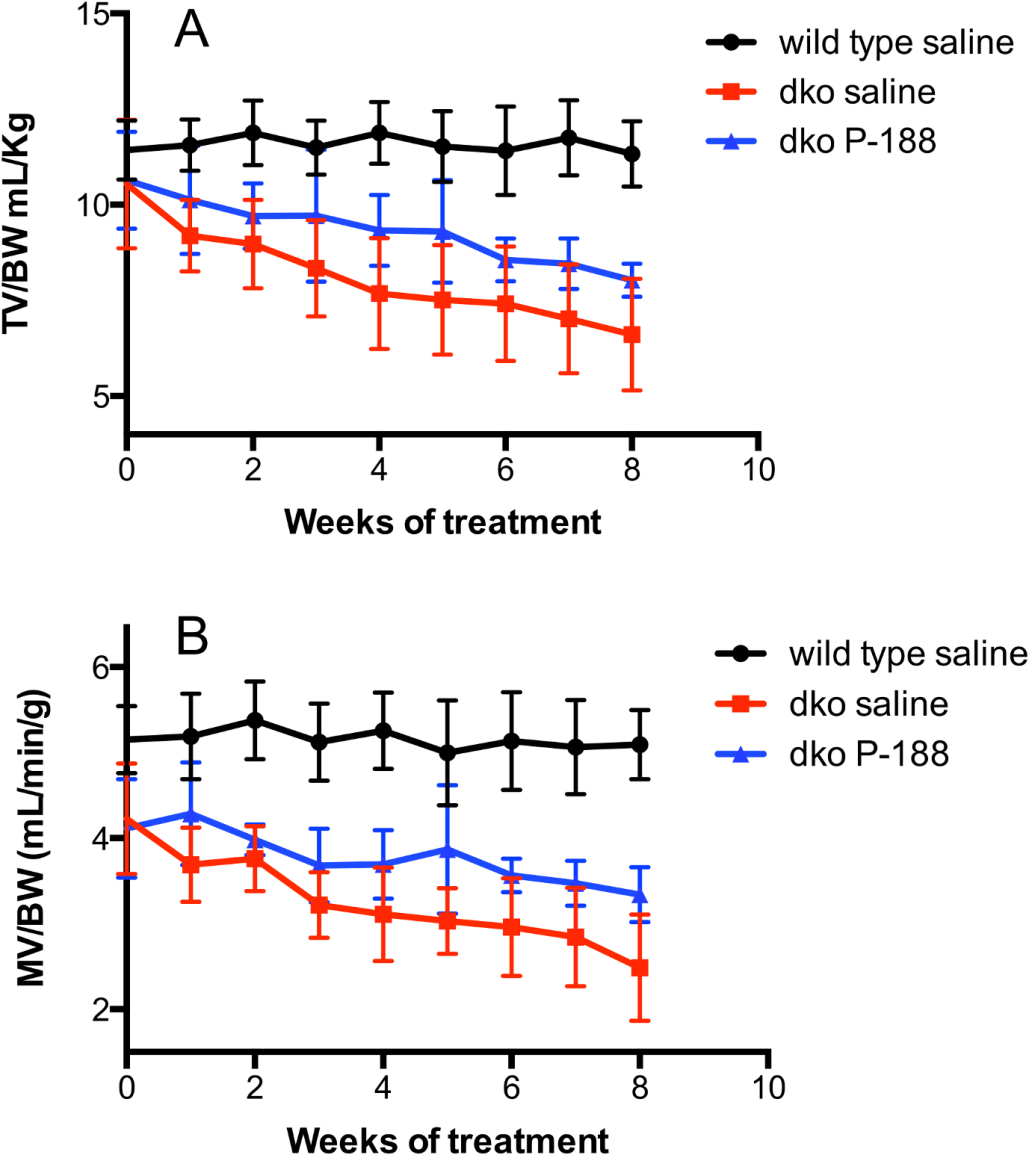
Effects of NF treatment on TV/BW and MV/BW in dko mice over time. Dko mice were treated with 1 mg/Kg of P-188 NF QD, s.c, from ages 3–11 weeks. The black line represents wild-type mice (C57BL/10 SnJ) treated with saline; the red line represents *dko* mice treated with saline; the blue line represents dko treated with 1 mg/Kg P-188 NF. TV/BW and MV/BW are shown in panels A, and B, respectively. Data points are means +/- S.D. N = 8/group wild type and 5/group dko saline and dko P-188 NF. For panels A and B, P < 0.0001 for dko 1 mg/Kg P-188 NF group vs. dko saline and wild type saline.

**Fig 3:**
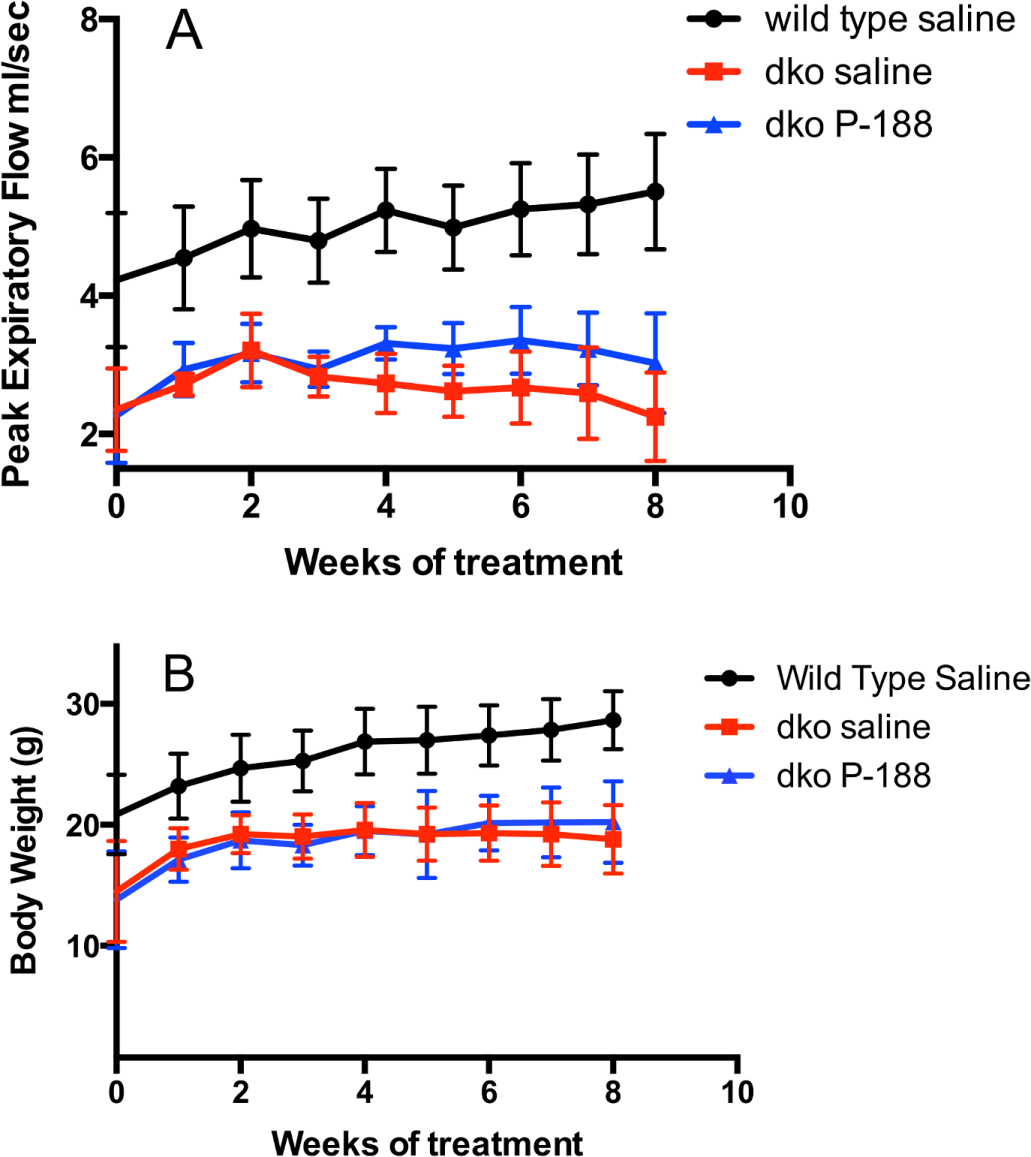
Effects of NF treatment on PEF and BW in dko mice over time. Dko mice were treated with 1 mg/Kg of P-188 NF QD, s.c, from ages 3–11 weeks. The black line represents wild-type mice (C57BL/10 SnJ) treated with saline; the red line represents *dko* mice treated with saline; the blue line represents dko treated with 1 mg/Kg P-188 NF. PEF and BW are shown in panels A & B, respectively. P < 0.0001 for wild type saline vs. all other groups. For panel A, P = 0.01 to 0.001 for dko 1 mg/Kg P-188 NF group vs. dko saline. For panel B, there is no significant difference between dko groups treated with saline or P-188 NF.

Three week-old dko mice had demonstrable respiratory deficits at baseline (S1 Table). The overall effects of 8 weeks of P-188 NF or prednisone treatment at 1 mg/Kg on respiration in the dko mouse model are shown in Figs 2 and 3, Table 5 and S12–S14 Figs. Compared with the saline-treated dko group, the P-188 NF treated group showed significant effects in 9 out of the 12 respiratory parameters. Prednisone treatment affected the same parameters with the exception of peak expiratory flow (PEF). Where affects were observed, they were similar for both treatment groups (Table 3). Most of the effects observed with P-188 NF can be characterized as a decrease in the rate of decline (TV, MV, TV/BW, MV/BW), or a stabilization (F, Penh, Rpef, PEF) of the respiratory parameter (Figs 2 and 3 and S12–S14). Similar effects were seen with prednisone. Since these studies were performed on mice starting at 3 weeks of age, we observed improvements in respiratory function for the first 2 weeks of each study in the dko saline and P-188 NF or prednisone treated groups that were likely due to maturation of the mice and not treatment. Either P-188 NF or prednisone treatment significantly improved respiratory parameters above what was seen at the 2 weeks of treatment time point.

**Table 5:** Overall effect of P-188 NF and prednisone at 1 mg/Kg versus saline on respiratory parameters in the dko mouse model.

Survivors	F	TV	TB/BW	MV	MV/BW	Penh	Rpef	PIF	PEF	Ti	Te	BW
P-188 (N = 9)	*	***	****	****	****	**	NS	**	***	*	NS	NS
Pred. (N = 6)	*	***	***	*	****	**	NS	***	NS	*	NS	NS

Two-way ANOVA (treatment vs. time) vs. dko saline (main treatment effect) post-hoc test.

* P< 0.05.

** P = 0.01 to 0.001.

*** P < 0.001.

**** P < 0.0001.

These results demonstrate that P-188 NF administered at 3 weeks of age in the dko mouse model can significantly delay the respiratory decline and the magnitude of the response on multiple parameters suggesting that P-188 NF has a beneficial impact on overall respiratory function.

### Histology

#### Analysis of centralized nuclei

**Fig 4:**
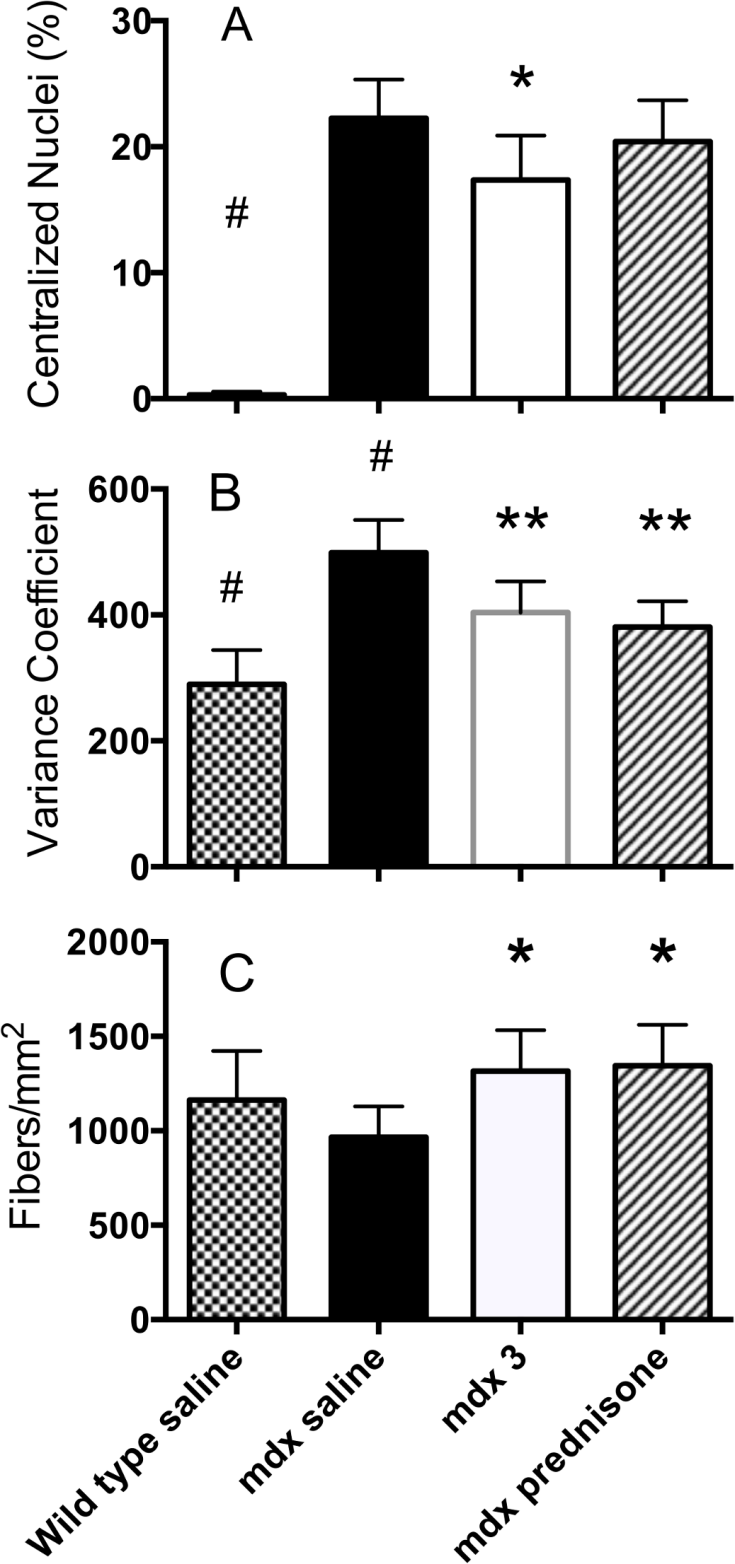
Histological parameters from diaphragm muscle from saline, P-188 NF, or prednisone treated *mdx* mice at 1 year of age. *Mdx* and wild type control mice were age to seven months and then randomized into groups (N = 12) based on tidal volume measurements. Mice were treated QD by s.c injection with saline, P-188 NF at 3 mg/Kg or prednisone (1 mg/Kg). At termination, diaphragm muscle was harvested, washed and embedded in paraffin. Sections from across each diaphragm were stained with wheat germ agglutinin and DAPI (Panels A & C) or lectin antibody (Panel B) and photographed. Images were analyzed using Nikon Elements software, or by direct counting. (A) Percentage of fibers with centralized nuclei; (B) Variance coefficient of the mean minimum Ferets diameter of diaphragm fibers; and (C) Fiber density. A minimum of 10,000 fibers was assessed for each parameter. * < 0.001 vs. *mdx* saline, ^#^ P < 0.001 vs. all other groups.

**Fig 5:**
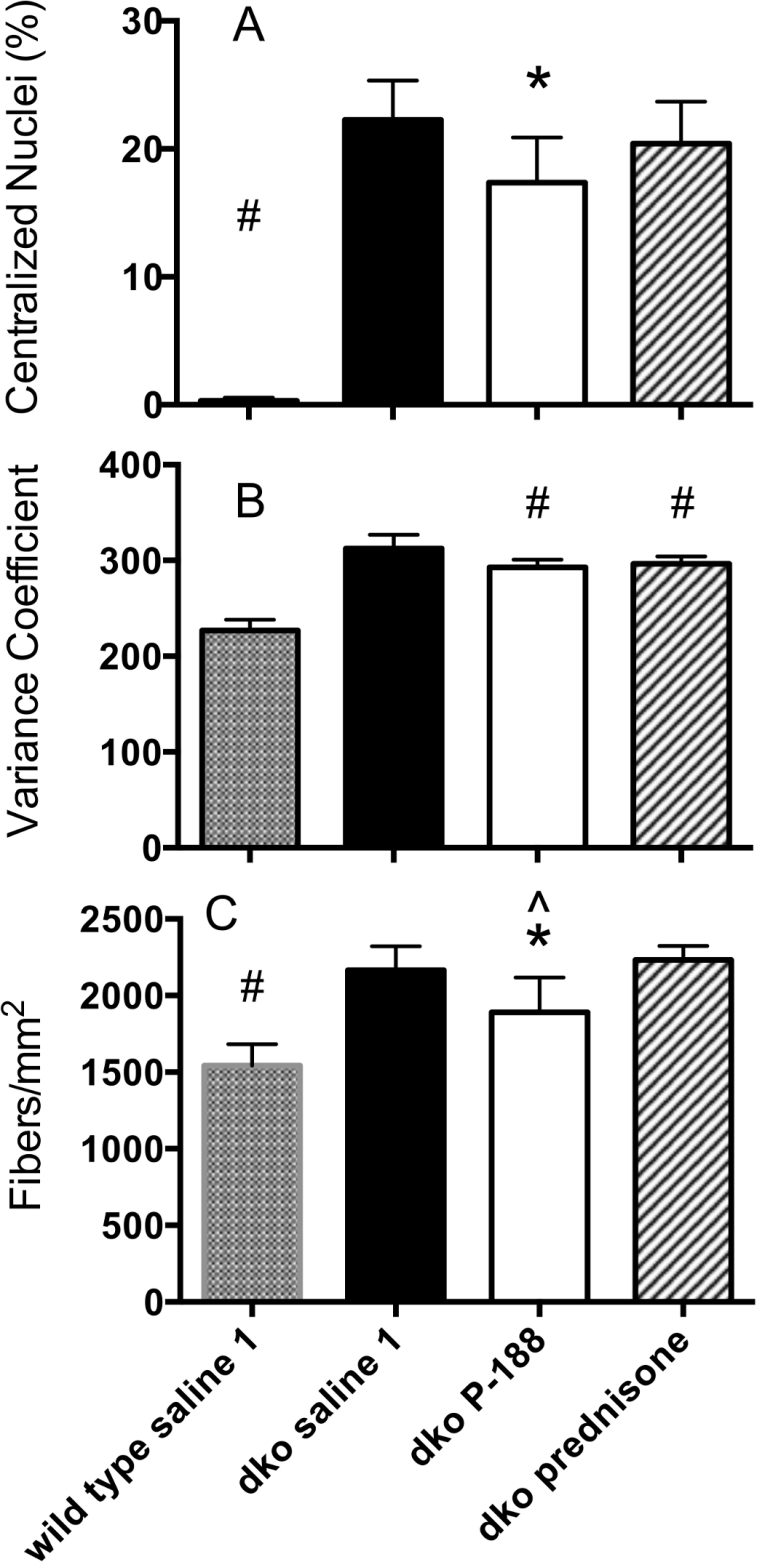
Histological parameters from diaphragm muscle from saline, P-188 NF, or prednisone treated dko mice at 11 weeks of age. Wild type or dko mice were treated with saline, P-188 NF (1 mg/Kg) or prednisone (1 mg/Kg) QD, s.c. for 8 weeks beginning at 3 weeks of age. At termination, diaphragm muscle was harvested, washed and embedded in paraffin. Sections from across each diaphragm were stained with wheat germ agglutinin and DAPI (Panels A & C) or lectin antibody (Panel B) and photographed. Images were analyzed using Nikon Elements software, or by direct counting. (A) Percentage of fibers with centralized nuclei; (B) Variance coefficient of the mean minimum Ferets diameter of diaphragm fibers; and (C) Fiber density. * < 0.01 vs. dko saline, ** P < 0.001 vs. dko saline, ^#^ P < 0.001 vs. all other groups.

The absence of dystrophin in muscle cells disrupts the dystrophin glycoprotein complexes (DGC) and leads to uncontrolled influx of calcium. This triggers a set of pathological processes that lead to degradation of muscle cell proteins, cell damage and muscle wasting. The newly differentiated muscle fibers exhibit centrally located nuclei and heterogeneity of fiber size [33, 34]. The percentage of fibers with centralized nuclei (CN) from the diaphragm of each animal in the *mdx* study was determined (Fig 4, Panel A). Cells from the *mdx* saline group exhibited centralized nuclei in ~23% of the fibers compared to <1% in the wild-type-saline group. P-188 NF or prednisone treatment significantly reduced the percentage of fibers containing CN in *mdx* mice. The 3 mg/Kg P-188 NF and prednisone groups showed a percentage of CN of 12% and 14.4%, respectively. A decrease in the percentage of centralized nuclei was also seen in dko mice treated with either P-188 NF (22% lower) or prednisone (10% lower) (Fig 5, Panel A).

The results from these 2 studies indicate a slower rate of degeneration/regeneration in P-188 NF- and prednisone-treated *mdx* and dko mice compared to saline-treated *mdx* mice.

#### Analysis of fiber size and density

When skeletal muscle fibers are lost through degeneration they are replaced by a regenerative process until depletion of satellite cells. The cross-sectional area of regenerating or recently regenerated fibers is expected to be smaller and more variable than that of mature muscle fibers. To determine the variability, the variance coefficient of the Feret’s diameter of muscle fibers was determined in 4–5 cross sections from each *mdx* mouse diaphragm representing different sections from across the length of muscle. The results are shown in Fig 4, Panel B and Fig 5 Panel B. In the *mdx* mouse study, the VC calculated for diaphragms of the wild type mouse group was 280, which is in line with that reported for non-dystrophic mice (VC = 237) [33]. The *mdx* saline-treated group had a VC of 465, which is similar to that reported previously for 7-week-old *mdx* mouse diaphragm muscle (VC = 402) [33]. Treatments that prevent or slow degeneration are expected to bring the *mdx* VC closer to the wild type VC. It can be seen in Fig 4B that treatment with 3 mg/Kg of P-188 NF significantly shifted the *mdx* VC lower (380) towards that of the wild type mouse diaphragms. The same effect was seen with prednisone treatment. There was no statistical difference between the P-188 NF doses and prednisone. In addition, treatment of *mdx* mice with either dose of P-188 NF or prednisone increased the number of fibers per unit area of diaphragm muscle (Fig 4, Panel C).

In the dko mouse studies, the wild type mouse VC values were in line with those seen in the *mdx* studies. However, the VC of the minimum Feret’s diameter of 11-week old dko mice treated with saline was not as high as in saline-treated *mdx* mice at 1 year of age indicating less variability in fiber size (Fig 5, Panel B). This might be expected if the muscle fibers of the 11-week old dko mice regenerate efficiently. P-188 NF or prednisone treatment resulted in a small but significant decrease in VC compared to dko mice treated with saline (Fig 5, Panel B). This indicates muscle fibers in the diaphragms of mice from both treatment groups are less heterogeneous in size. Muscle fiber densities of the dko mouse groups were significantly higher than those in the wild type groups (Fig 5, Panel C) but there was no difference between the dko saline and dko P-188 NF or prednisone-treated groups. In the dko study evaluating prednisone, the fiber density in the dko mouse groups was significantly greater than those groups in the P-188 NF study. The reason for this difference is unknown. However the mice in these studies were not from the same breeding pairs, which might contribute to the difference.

**Fig 6:**
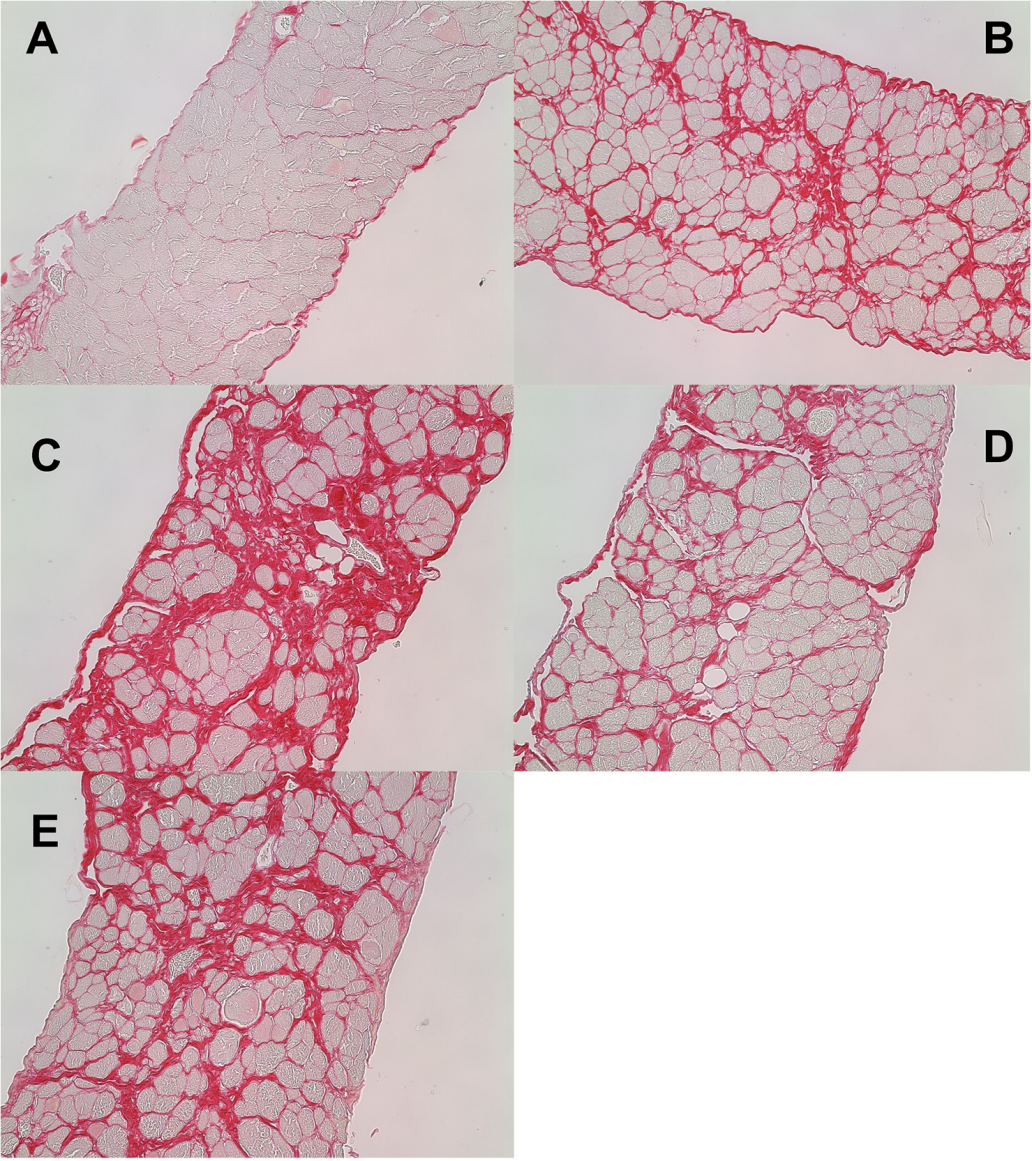
Fibrosis of diaphragm muscle from 1-year-old *mdx* and wild type control mice after 22 weeks of treatment with saline or P-188 NF. Sections of diaphragms from the same groups of mice described in Fig 1 were stained with Picosirius Red to visualize collagen deposition. All staining was done on diaphragm muscle from 12-month old mice with the exception of Panel B. Shown in the figure are cross sections of diaphragm muscle from: Panel A, wild type control saline-treated mouse; Panel B, a 7 month old *mdx* mouse; Panel C, an *mdx* saline-treated mouse; Panel D, an *mdx* mouse treated with 3 mg/Kg P-188 NF; Panel E, an *mdx* mouse treated with 1 mg/Kg prednisone.

**Fig 7:**
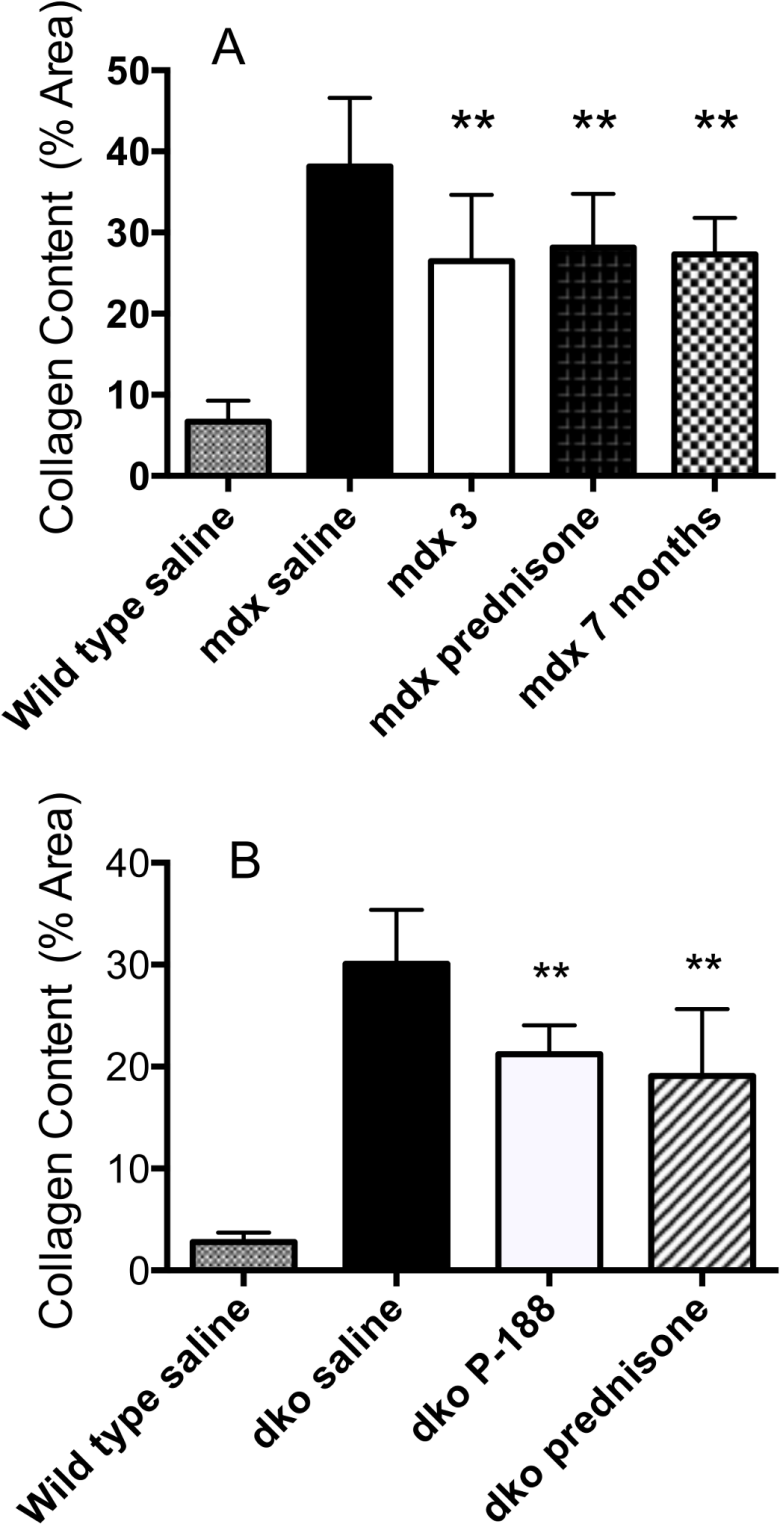
Effect of P-188 NF on collagen composition in diaphragm muscle. Measurements of picosirius red staining were determined from in *mdx* (panel A) and dko mice (panel B) at the end of the respective treatment periods. All surviving mice were use in the analysis. Staining was quantified using Nikon Elements software to determine the percentage of area stained in each section. A minimum of 6 sections/diaphragm was assessed. The wild type saline group was significantly different from all other groups, ^#^P < 0.0001. Panel A, ^**^ P <0.01 vs. *mdx* saline; Panel B ** P < 0.01 vs. dko saline.

H&E staining of longitudinal sections of diaphragm muscle from wild type and mdx mice revealed many features of muscle damage (S15 Fig). These included centralized nuclei, mononuclear cell accumulation, necrotic fibers, and vesicles that are presumed to be lipid vesicles. The diaphragm from the mdx mouse treated with saline at 12 months (S15C Fig) exhibits paler fibers than the other sections indicating more fibrosis, which was confirmed with Picosirius red staining (see Figs 6 and 7).

#### Analysis of fibrosis

Representative cross sections of diaphragm muscle from *mdx* mice muscle, stained with Picosirius Red to show collagen, are shown in Fig 6. Panels A-E show wild type (saline), *mdx* at 7 months of age, *mdx* (saline) at 12 months of age, *mdx* P-188 NF and *mdx* prednisone treated groups at 12 months of age respectively. There was minor collagen deposition in the diaphragms from wild type mice at 12 months of age (Fig 6, Panel A). However, there was significant collagen deposition in the *mdx* mouse diaphragm at 7 months of age (Fig 6, Panel B) and remarkably more at 12 months (Fig 6, Panel C). The measureable collagen deposition in the diaphragm muscle of the *mdx* mice at 7 months of age was expected [28, 29]. Each of the treatment groups had much less collagen staining than the *mdx* saline-treated group at 12 months and look similar to that observed in 7 month old *mdx* mice, the age when treatment was initiated (Panels D-F). Quantitative measurement of the extent of fibrosis in *mdx* mice, measured as the percentage of red area/total area is shown in Fig 7, Panel A. The wild-type group showed 6.6% staining and was significantly different from all other groups (P < 0.0001). The 7-month *mdx* mouse group had 27.3% fibrotic area compared to 38% for the 12-month old saline treated group indicating a significant (P < 0.0001) increase in fibrosis during the study period. Treatment with either P-188 NF or prednisone resulted in a significantly lower level percentage of collagen in the cross sections of 26 and 28% for P-188 NF and prednisone, respectively, measured at 12 months of age. The 3 mg/Kg P-188 NF group had significantly less fibrosis than either the prednisone or 7 month-old *mdx* groups. There was no difference between the 7 month-old *mdx* group and the prednisone- treated group.

Diaphragm muscles from dko mice treated with P-188 NF or prednisone at 1 mg/Kg also showed a substantial decrease in fibrosis (Fig 6B), approximately a 33% reduction compared with their respective dko saline controls. These results demonstrate that P-188 NF treatment has the potential to slow or stop the deposition of collagen in the diaphragm.

## Discussion

The studies reported here were undertaken to test the hypothesis that P-188 NF treatment would have functional and structural effects on contraction-induced membrane damage in diaphragm muscle. We observed significant differences in several respiratory parameters between groups of *mdx* mice treated for 22 weeks (Table 2) of which TV/BW and MV/BW ratio were clearly biologically meaningful (Figs 1 and S11). TV/BW was improved 12% by P-188 NF treatment compared with saline-treated *mdx* mice or compared to baseline. P-188 NF treatment also significantly impacted diaphragm structure in the 1-year-old *mdx* mice suggesting that P-188 NF treatment protected muscle fibers from degeneration. When we switched to a more severe muscular dystrophy model the biological effects of P-188 NF treatment were even more evident. There was a demonstrable slowing of the loss of respiratory function in dko mice treated for 8 weeks with P-188 NF (Table 3, and S12–S14 Figs). Maintenance of respiratory function was associated with a diaphragm muscle protective effect of P-188 NF treatment. These studies support our hypothesis that P-188 NF can protect from contraction-induced diaphragm membrane damage. It is also of note that the significant changes observed in the *mdx* and dko mouse studies occurred in animals with established respiratory dysfunction indicating that P-188 NF has the potential to treat, rather than just prevent, respiratory dysfunction.

Prednisone was run as a positive control in these studies. Corticosteroid therapy has been shown to delay the loss of respiratory function in DMD patients [6, 48]. The results shown here indicate that, in these 2 mouse models of muscular dystrophy, the activity of prednisone is similar to that of P-188 NF both qualitatively and quantitatively. The similarity between the effects of P-188 treatment and this standard of care therapy in *mdx* and dko mice is exciting since it suggests that P-188 NF might have similar positive effects in patients. With a better side effect profile than prednisone P-188 NF might be an alternative therapy for the many DMD patients, for whom the side effects of corticosteroid treatment are severe and unacceptable. In addition, P-188 NF treatment effects might be additive to those of corticosteroid treatment given the difference in their proposed mechanisms of action. Additional work will be needed to demonstrate the effects of combination P-188-prednisone treatment. While we don’t know if the functional effects in *mdx* mice will translate into functional improvements in DMD patients, the fact that nine of twelve respiratory parameters were affected in the same fashion by P-188 NF and prednisone treatment provides a basis for suggesting that P-188 NF might also delay loss of respiratory function in patients.

Our results showing effects of P-188 NF on respiratory function, through protection of diaphragm muscle, are in contrast to *in situ* results reported recently by Terry et al. [24] that show a modest drop in maximum force generated in Tibialis Anterior muscle. While the reason for the functional discrepancy is not known, we only evaluated diaphragm muscle so no direct comparison can be made. However, work by Dr. Joseph Metzger (Chair of the Phrixus Scientific Advisory Board) and colleagues have demonstrated that the route of administration of P-188 is critical to its pharmacodynamic properties with the s.c but not the i.p. route of administration conferring protection to dystrophic skeletal muscle [49]. The work presented here was performed administering P-188 s.c. while Terry et al. [24] administered P-188 i.p. The i.p. route of administration was also used in another paper that claimed that P-188 did not protect skeletal muscle [21]. Further, the Terry et al. study used Hypnorm and midazolam as anesthesia during functional evaluation. Both fentanyl (Hypnorm component) and P-188 have surfactant properties [50, 51] and have been shown to directly influence membrane structure and function [14, 52–58]. In addition, it is clear from a pharmacokinetic study that P-188 enhances the bioavailability of fentanyl from an oral transmucosal lozenge suggesting an interaction between these molecules at the membrane level [59]. The potential for interactions between these agents was not controlled for in the Terry et al. study [24]. Additional work will be required to determine if route of administration or choice of anesthesia contributed to these differences.

The histology results provide a mechanistic explanation for P-188 NF’s functional benefits. We hypothesized that P-188 NF protects diaphragm muscle fibers from membrane damage thereby decreasing the amount of regeneration taking place at any specific time. The reduced need for regenerative capacity likely preserves that capacity for a longer period. Diaphragm regeneration, as measured by centralized nuclei, has been reported to be above 40% at 20 weeks of age [60] and above 40% at 4 and 7 months of age [61] in *mdx* mice. The 26% level seen in the 1 year old *mdx* mice treated with saline (Fig 3) suggests that the active regenerative process in diaphragm muscle may be slowing compared to that in younger mice. Indeed, 16-month-old mice have been reported to have 14% centralized nuclei [62] supporting this contention. In any case, the results in Fig 3 demonstrate that diaphragm muscle has reduced regenerative capacity in 1-year-old *mdx* mice.

The results presented here demonstrate that daily, low dose, subcutaneous administration of P-188 NF can improve respiratory function in the *mdx* mouse model. Since respiratory failure is the leading cause of death in the DMD population, P-188 NF could represent an important new therapy to slow the loss of respiratory function and potentially increase life span in DMD patients.

Wild type and dko mice (3 weeks of age) were randomized into groups and evaluated for respiratory activity using un-anesthetized, un-restrained whole body plethysmography. This table shows the baseline readings for each respiratory parameter evaluated.

## Supporting Information

S1 Fig Dose-range finding study with P-188 NF in a rat model of heart failure. Myocardial infarction was induced in Sprague-Dawley rats by complete ligation of the left anterior descending coronary artery and significant heart failure developed over 8-weeks of incubation as determined by echocardiography (ejection fraction < 30%) (CHF rats). Ejection fraction was measured at baseline and 2 days later the CHF rats were dosed i.v., by tail vein injection, with 1.5 mg/Kg of P-188 NF and echoed 4 hr post dose. After a 3 day washout period, the rats were dosed with 4.6 mg/kg of P-188 NF and echoed 4 hr post dose. Ejection fraction was increased at both doses of P-188. One rat in the 4.6 mg/Kg group did not respond keeping value at this dose low.
(TIF)

S2 Fig Effects of P-188 NF and prednisone on respiration rate in *mdx* mice over time. *Mdx* mice were treated QD, *s*.*c*. with P-188 NF or prednisone from age 7 months to 12 months ± 2 weeks. The black line represents wild-type mice (C57BL/10 SnJ) treated with saline. The red line represents *mdx* mice treated with saline. The blue line represents mice treated with 3 mg/Kg P-188 NF (Panel A) or 1 mg/Kg prednisone (Panel B). Data points are means +/- S.D. N = 12/group for both groups, except N = 11 for mdx 3 mg/Kg P-188 NF at 20 and 22 weeks. P < 0.0001 for wild type saline vs. all mdx groups. Panel A, P < 0.0001 for mdx 3 mg/Kg group vs. mdx saline. Panel B. P < 0.05 for mdx 1 mg/Kg prednisone group vs. mdx saline.
(TIF)

S3 Fig Effects of P-188 NF and prednisone on Tidal Volume in the *mdx* mouse over time. *Mdx* mice were treated QD, *s*.*c*. with P-188 NF or prednisone from age 7 months to 12 months ± 2 weeks. The black line represents wild-type mice (C57BL/10 SnJ) treated with saline. The red line represents *mdx* mice treated with saline. The blue line represents mice treated with 3 mg/Kg P-188 NF (Panel A) or 1 mg/Kg prednisone (Panel B). Data points are means +/- S.D. N = 12/group for both groups, except N = 11 for mdx 3 mg/Kg P-188 at 20 and 22 weeks. P < 0.0001 for wild type saline vs. all mdx groups. Panel A, P < 0.001 for mdx 3 mg/Kg group vs. wild type saline. Panel B. P < 0.001 for the mdx 1 mg/Kg prednisone group vs. mdx saline.
(TIF)

S4 Fig Effects of P-188 NF and prednisone on Minute Volume in the *mdx* mouse over time. *Mdx* mice were treated QD, *s*.*c*. with P-188 NF or prednisone from age 7 months to 12 months ± 2 weeks. The black line represents wild-type mice (C57BL/10 SnJ) treated with saline. The red line represents *mdx* mice treated with saline. The blue line represents mice treated with 3 mg/Kg P-188 NF (Panel A) or 1 mg/Kg prednisone (Panel B). Data points are means +/- S.D. N = 12/group for both groups, except N = 11 for mdx 3 mg/Kg P-188 at 20 and 22 weeks. P < 0.0001 for wild type saline vs. all mdx groups. Panel A, P < 0.0001 for mdx 3 mg/Kg group vs. mdx saline. Panel B. P < 0.05 for the mdx 1 mg/Kg prednisone group vs. mdx saline. P < 0.01 for the mdx P-188 NF group vs. the mdx prednisone group.
(TIF)

S5 Fig Effects of P-188 NF and prednisone on Enhanced Pause in the *mdx* mouse over time. *Mdx* mice were treated QD, *s*.*c*. with P-188 NF or prednisone from age 7 months to 12 months ± 2 weeks. The black line represents wild-type mice (C57BL/10 SnJ) treated with saline. The red line represents *mdx* mice treated with saline. The blue line represents mice treated with 3 mg/Kg P-188 NF (Panel A) or 1 mg/Kg prednisone (Panel B). Data points are means +/- S.D. N = 12/group for both groups, except N = 11 for mdx 3 mg/Kg P-188 at 20 and 22 weeks. P < 0.0001 for wild type saline vs. all mdx groups. Panel A, P < 0.01 for mdx 3 mg/Kg group vs. mdx saline. Panel B. P < 0.0001 for the mdx 1 mg/Kg prednisone group vs. mdx saline.
(TIF)

S6 Fig Effects of P-188 NF and prednisone on Rpef in the *mdx* mouse over time. *Mdx* mice were treated QD, *s*.*c*. with P-188 NF or prednisone from age 7 months to 12 months ± 2 weeks. The black line represents wild-type mice (C57BL/10 SnJ) treated with saline. The red line represents *mdx* mice treated with saline. The blue line represents mice treated with 3 mg/Kg P-188 NF (Panel A) or 1 mg/Kg prednisone (Panel B). Data points are means +/- S.D. N = 12/group for both groups, except N = 11 for mdx 3 mg/Kg P-188 at 20 and 22 weeks. P < 0.0001 for the wild type saline groups vs. mdx saline and mdx P-188 NF. The wild type saline and mdx prednisone groups were not significantly different. Panel A, P < 0.0001 for mdx 3 mg/Kg group vs. mdx saline. Panel B. P < 0.0001 for the mdx 1 mg/Kg prednisone group vs. mdx saline. P < 0.05 for mdx P-188 NF vs. mdx prednisone.
(TIF)

S7 Fig Effects of P-188 NF and prednisone on Peak Inspiratory Flow in the *mdx* mouse over time. *Mdx* mice were treated QD, *s*.*c*. with P-188 NF or prednisone from age 7 months to 12 months ± 2 weeks. The black line represents wild-type mice (C57BL/10 SnJ) treated with saline. The red line represents *mdx* mice treated with saline. The blue line represents mice treated with 3 mg/Kg P-188 NF (Panel A) or 1 mg/Kg prednisone (Panel B). Data points are means +/- S.D. N = 12/group for both groups, except N = 11 for mdx 3 mg/Kg P-188 at 20 and 22 weeks. P < 0.0001 for wild type saline vs. all mdx groups. Panel A, P < 0.0001 for mdx 3 mg/Kg group vs. wild type saline. Panel B. P < 0.0001 for the mdx 1 mg/Kg prednisone group vs. mdx saline.
(TIF)

S8 Fig Effects of P-188 NF and prednisone on Peak Expiratory Flow in the *mdx* mouse over time. *Mdx* mice were treated QD, *s*.*c*. with P-188 NF or prednisone from age 7 months to 12 months ± 2 weeks. The black line represents wild-type mice (C57BL/10 SnJ) treated with saline. The red line represents *mdx* mice treated with saline. The blue line represents mice treated with 3 mg/Kg P-188 NF (Panel A) or 1 mg/Kg prednisone (Panel B). Data points are means +/- S.D. N = 12/group for both groups, except N = 11 for mdx 3 mg/Kg P-188 at 20 and 22 weeks. P < 0.0001 for wild type saline vs. all mdx groups. There was no significant difference between mdx groups.
(TIF)

S9 Fig Effects of P-188 NF and prednisone on Inspiration Time in the *mdx* mouse over time. *Mdx* mice were treated QD, *s*.*c*. with P-188 NF or prednisone from age 7 months to 12 months ± 2 weeks. The black line represents wild-type mice (C57BL/10 SnJ) treated with saline. The red line represents *mdx* mice treated with saline. The blue line represents mice treated with 3 mg/Kg P-188 NF (Panel A) or 1 mg/Kg prednisone (Panel B). Data points are means +/- S.D. N = 12/group for both groups, except N = 11 for mdx 3 mg/Kg P-188 at 20 and 22 weeks. P < 0.0001 for wild type saline vs. all mdx groups. Panel A, P < 0.01 for mdx 3 mg/Kg group vs. mdx saline. Panel B. P < 0.01 for the mdx 1 mg/Kg prednisone group vs. mdx saline.
(TIF)

S10 Fig Effects of P-188 NF and prednisone on Expiration Time in the *mdx* mouse over time. *Mdx* mice were treated QD, *s*.*c*. with P-188 NF or prednisone from age 7 months to 12 months ± 2 weeks. The black line represents wild-type mice (C57BL/10 SnJ) treated with saline. The red line represents *mdx* mice treated with saline. The blue line represents mice treated with 3 mg/Kg P-188 NF (Panel A) or 1 mg/Kg prednisone (Panel B). Data points are means +/- S.D. N = 12/group for both groups, except N = 11 for mdx 3 mg/Kg P-188 at 20 and 22 weeks. P < 0.01 for wild type saline vs. all mdx P-188 NF and P < 0.0001 vs. mdx prednisone. Panel A, Not significant for mdx 3 mg/Kg group vs. mdx saline. Panel B. P < 0.05 for the mdx 1 mg/Kg prednisone group vs. mdx saline.
(TIF)

S11 Fig Effects of P-188 NF and prednisone on MV/BW in the *mdx* mouse over time. *Mdx* mice were treated QD, *s*.*c*. with P-188 NF or prednisone from age 7 months to 12 months ± 2 weeks. The black line represents wild-type mice (C57BL/10 SnJ) treated with saline. The red line represents *mdx* mice treated with saline. The blue line represents mice treated with 3 mg/Kg P-188 NF (Panel A) or 1 mg/Kg prednisone (Panel B). Data points are means +/- S.D. N = 12/group for both groups, except N = 11 for mdx 3 mg/Kg P-188 at 20 and 22 weeks. P < 0.0001 for wild type saline vs. all mdx P-188 NF, P < 0.05 for mdx saline vs. mdx P-188 and P < 0.001 for mdx saline vs. mdx prednisone.
(TIF)

S12 Fig Effects of P-188 treatment on F, TV and MV in dko mice over time. Dko mice were treated with 1 mg/Kg of P-188 NF once per day, s.c, for 8 weeks from ages 3–11 weeks. Respiration was measured weekly by WBP. The key on the right side of each panel identifies the groups and the parameter is indicated on the Y-axis. Data points are means +/- S.D. N = 8/group wild type and 5/group dko saline and dko P-188. P < 0.0001 for wild type saline vs. All mdx groups. Panel A, P < 0.05 for mdx P-188 NF vs. mdx saline. Panel B, P < 0.001 for mdx P-188 NF vs. mdx saline. Panel C, P < 0.0001 001 for mdx P-188 NF vs. mdx saline. N = 18 for wild type saline and N = 9 for mdx P-188 NF.
(TIF)

S13 Fig Effects of P-188 treatment on Penh, Rpef and PIF in dko mice over time. Dko mice were treated with 1 mg/Kg of P-188 NF once per day, s.c, for 8 weeks from ages 3–11 weeks. Respiration was measured weekly by WBP. The key on the right side of each panel identifies the groups and the parameter is indicated on the Y-axis. Data points are means +/- S.D. N = 8/group wild type and 9/group dko saline and dko P-188. P < 0.0001 for wild type saline vs. All mdx groups. The significance for any differences observed can be found in Table 3 in the P-188 (N = 5) row. Panel A, P < 0.01 for mdx P-188 NF vs. mdx saline. Panel B, Not significant for mdx P-188 NF vs. mdx saline. Panel C, P < 0.05 for mdx P-188 NF vs. mdx saline. N = 18 for wild type saline and N = 9 for mdx P-188 NF.
(TIF)

S14 Fig Effects of P-188 treatment on Ti, and Te in dko mice over time. Dko mice were treated with 1 mg/Kg of P-188 NF once per day, s.c, for 8 weeks from ages 3–11 weeks. Respiration was measured weekly by WBP. The key on the right side of each panel identifies the groups and the parameter is indicated on the Y-axis. Data points are means +/- S.D. N = 8/group wild type and 9/group dko saline and dko P-188. P < 0.0001 for wild type saline vs. All mdx groups. The significance for any differences observed can be found in Table 3 in the P-188 (N = 5) row. Panel A, P < 0.05 for mdx P-188 NF vs. mdx saline. Panel B, Not significant for mdx P-188 NF vs. mdx saline.
(TIF)

S15 Fig H&E stained longitudinal sections from 1-year-old *mdx* and wild type control mice after 22 weeks of treatment with saline or P-188 NF. Sections of diaphragms from the same groups of mice described in Fig 1 were stained with H&E. All staining was done on diaphragm muscle from 12-month old mice with the exception of Panel B, which shows a diaphragm from a 7 month old untreated mdx mouse. Shown in the figure are longitudinal sections of diaphragm muscle from: Panel A, wild type control saline-treated mouse; Panel B, a 7 month old mdx mouse; Panel C, an *mdx* saline-treated mouse; Panel D, an *mdx* mouse treated with 3 mg/Kg P-188 NF; Panel E, an *mdx* mouse treated with 1 mg/Kg prednisone.
(TIF)

S1 Table Comparison of baseline respiratory function and body weight between groups of wild type and dko mice at 3 weeks of ageg. (DOCX)
