# Supplementary figures and images for: Chronic Dosing with Membrane Sealant Poloxamer 188 NF Improves Respiratory Dysfunction in Dystrophic Mdx and Mdx/Utrophin-/- Mice
Source: PLoS One. 2015 Aug 6;10(8):e0134832. doi: 10.1371/journal.pone.0134832 (PMC4527695; doi:10.1371/journal.pone.0134832)

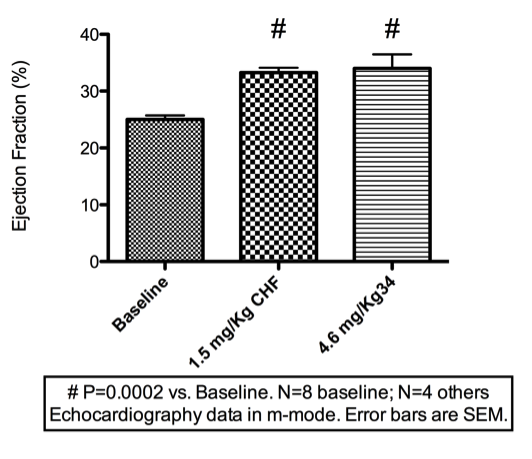

Supplement: S1 Fig — Myocardial infarction was induced in Sprague-Dawley rats by complete ligation of the left anterior descending coronary artery and significant heart failure developed over 8-weeks of incubation as determined by echocardiography (ejection fraction < 30%) (CHF rats). Ejection fraction was measured at baseline and 2 days later the CHF rats were dosed i.v., by tail vein injection, with 1.5 mg/Kg of P-188 NF and echoed 4 hr post dose. After a 3 day washout period, the rats were dosed with 4.6 mg/kg of P-188 NF and echoed 4 hr post dose. Ejection fraction was increased at both doses of P-188. One rat in the 4.6 mg/Kg group did not respond keeping value at this dose low. (TIF) [file pone.0134832.s001.tif]

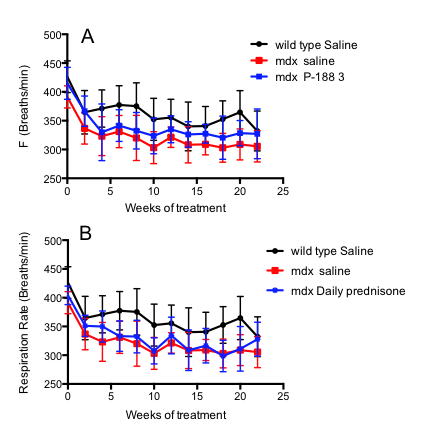

Supplement: S2 Fig — Mdx mice were treated QD, s.c. with P-188 NF or prednisone from age 7 months to 12 months ± 2 weeks. The black line represents wild-type mice (C57BL/10 SnJ) treated with saline. The red line represents mdx mice treated with saline. The blue line represents mice treated with 3 mg/Kg P-188 NF (Panel A) or 1 mg/Kg prednisone (Panel B). Data points are means +/- S.D. N = 12/group for both groups, except N = 11 for mdx 3 mg/Kg P-188 NF at 20 and 22 weeks. P < 0.0001 for wild type saline vs. all mdx groups. Panel A, P < 0.0001 for mdx 3 mg/Kg group vs. mdx saline. Panel B. P < 0.05 for mdx 1 mg/Kg prednisone group vs. mdx saline. (TIF) [file pone.0134832.s002.tif]

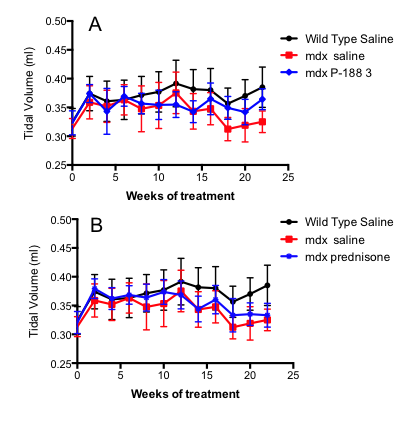

Supplement: S3 Fig — Mdx mice were treated QD, s.c. with P-188 NF or prednisone from age 7 months to 12 months ± 2 weeks. The black line represents wild-type mice (C57BL/10 SnJ) treated with saline. The red line represents mdx mice treated with saline. The blue line represents mice treated with 3 mg/Kg P-188 NF (Panel A) or 1 mg/Kg prednisone (Panel B). Data points are means +/- S.D. N = 12/group for both groups, except N = 11 for mdx 3 mg/Kg P-188 at 20 and 22 weeks. P < 0.0001 for wild type saline vs. all mdx groups. Panel A, P < 0.001 for mdx 3 mg/Kg group vs. wild type saline. Panel B. P < 0.001 for the mdx 1 mg/Kg prednisone group vs. mdx saline. (TIF) [file pone.0134832.s003.tif]

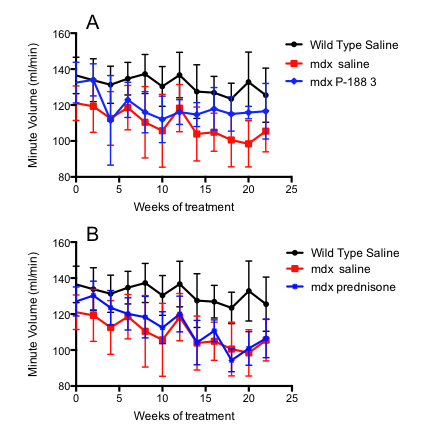

Supplement: S4 Fig — Mdx mice were treated QD, s.c. with P-188 NF or prednisone from age 7 months to 12 months ± 2 weeks. The black line represents wild-type mice (C57BL/10 SnJ) treated with saline. The red line represents mdx mice treated with saline. The blue line represents mice treated with 3 mg/Kg P-188 NF (Panel A) or 1 mg/Kg prednisone (Panel B). Data points are means +/- S.D. N = 12/group for both groups, except N = 11 for mdx 3 mg/Kg P-188 at 20 and 22 weeks. P < 0.0001 for wild type saline vs. all mdx groups. Panel A, P < 0.0001 for mdx 3 mg/Kg group vs. mdx saline. Panel B. P < 0.05 for the mdx 1 mg/Kg prednisone group vs. mdx saline. P < 0.01 for the mdx P-188 NF group vs. the mdx prednisone group. (TIF) [file pone.0134832.s004.tif]

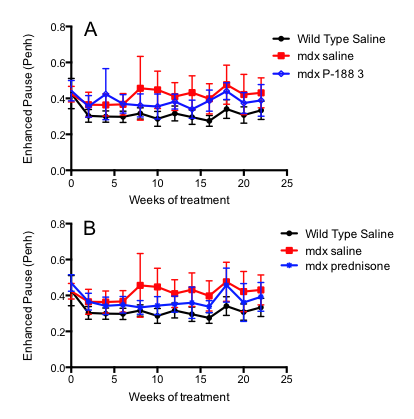

Supplement: S5 Fig — Mdx mice were treated QD, s.c. with P-188 NF or prednisone from age 7 months to 12 months ± 2 weeks. The black line represents wild-type mice (C57BL/10 SnJ) treated with saline. The red line represents mdx mice treated with saline. The blue line represents mice treated with 3 mg/Kg P-188 NF (Panel A) or 1 mg/Kg prednisone (Panel B). Data points are means +/- S.D. N = 12/group for both groups, except N = 11 for mdx 3 mg/Kg P-188 at 20 and 22 weeks. P < 0.0001 for wild type saline vs. all mdx groups. Panel A, P < 0.01 for mdx 3 mg/Kg group vs. mdx saline. Panel B. P < 0.0001 for the mdx 1 mg/Kg prednisone group vs. mdx saline. (TIF) [file pone.0134832.s005.tif]

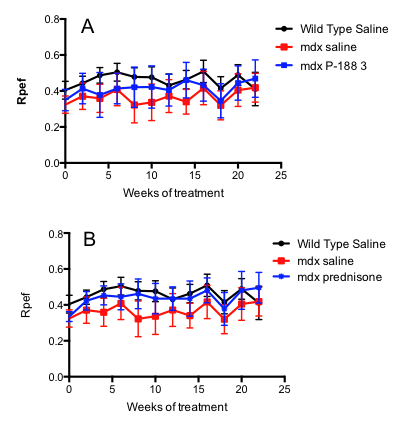

Supplement: S6 Fig — Mdx mice were treated QD, s.c. with P-188 NF or prednisone from age 7 months to 12 months ± 2 weeks. The black line represents wild-type mice (C57BL/10 SnJ) treated with saline. The red line represents mdx mice treated with saline. The blue line represents mice treated with 3 mg/Kg P-188 NF (Panel A) or 1 mg/Kg prednisone (Panel B). Data points are means +/- S.D. N = 12/group for both groups, except N = 11 for mdx 3 mg/Kg P-188 at 20 and 22 weeks. P < 0.0001 for the wild type saline groups vs. mdx saline and mdx P-188 NF. The wild type saline and mdx prednisone groups were not significantly different. Panel A, P < 0.0001 for mdx 3 mg/Kg group vs. mdx saline. Panel B. P < 0.0001 for the mdx 1 mg/Kg prednisone group vs. mdx saline. P < 0.05 for mdx P-188 NF vs. mdx prednisone. (TIF) [file pone.0134832.s006.tif]

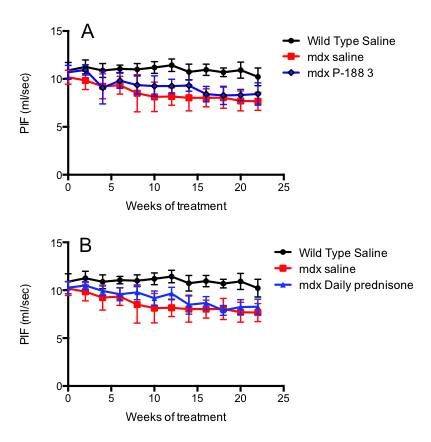

Supplement: S7 Fig — Mdx mice were treated QD, s.c. with P-188 NF or prednisone from age 7 months to 12 months ± 2 weeks. The black line represents wild-type mice (C57BL/10 SnJ) treated with saline. The red line represents mdx mice treated with saline. The blue line represents mice treated with 3 mg/Kg P-188 NF (Panel A) or 1 mg/Kg prednisone (Panel B). Data points are means +/- S.D. N = 12/group for both groups, except N = 11 for mdx 3 mg/Kg P-188 at 20 and 22 weeks. P < 0.0001 for wild type saline vs. all mdx groups. Panel A, P < 0.0001 for mdx 3 mg/Kg group vs. wild type saline. Panel B. P < 0.0001 for the mdx 1 mg/Kg prednisone group vs. mdx saline. (TIF) [file pone.0134832.s007.tif]

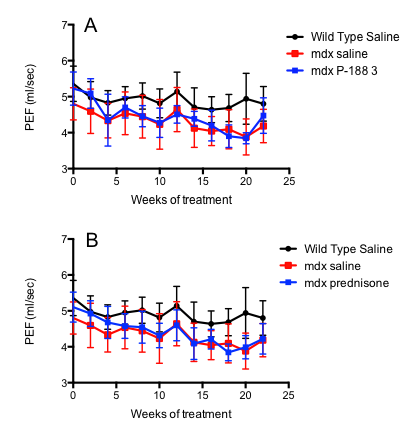

Supplement: S8 Fig — Mdx mice were treated QD, s.c. with P-188 NF or prednisone from age 7 months to 12 months ± 2 weeks. The black line represents wild-type mice (C57BL/10 SnJ) treated with saline. The red line represents mdx mice treated with saline. The blue line represents mice treated with 3 mg/Kg P-188 NF (Panel A) or 1 mg/Kg prednisone (Panel B). Data points are means +/- S.D. N = 12/group for both groups, except N = 11 for mdx 3 mg/Kg P-188 at 20 and 22 weeks. P < 0.0001 for wild type saline vs. all mdx groups. There was no significant difference between mdx groups. (TIF) [file pone.0134832.s008.tif]

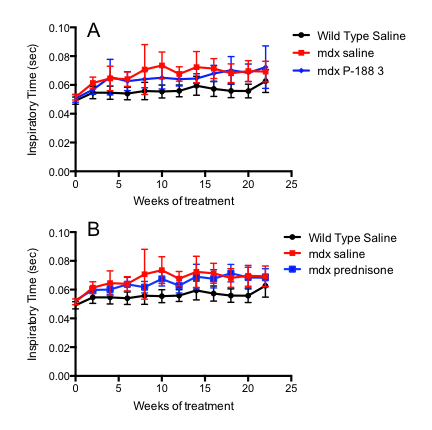

Supplement: S9 Fig — Mdx mice were treated QD, s.c. with P-188 NF or prednisone from age 7 months to 12 months ± 2 weeks. The black line represents wild-type mice (C57BL/10 SnJ) treated with saline. The red line represents mdx mice treated with saline. The blue line represents mice treated with 3 mg/Kg P-188 NF (Panel A) or 1 mg/Kg prednisone (Panel B). Data points are means +/- S.D. N = 12/group for both groups, except N = 11 for mdx 3 mg/Kg P-188 at 20 and 22 weeks. P < 0.0001 for wild type saline vs. all mdx groups. Panel A, P < 0.01 for mdx 3 mg/Kg group vs. mdx saline. Panel B. P < 0.01 for the mdx 1 mg/Kg prednisone group vs. mdx saline. (TIF) [file pone.0134832.s009.tif]

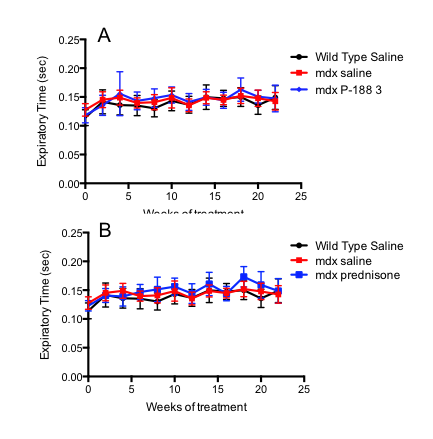

Supplement: S10 Fig — Mdx mice were treated QD, s.c. with P-188 NF or prednisone from age 7 months to 12 months ± 2 weeks. The black line represents wild-type mice (C57BL/10 SnJ) treated with saline. The red line represents mdx mice treated with saline. The blue line represents mice treated with 3 mg/Kg P-188 NF (Panel A) or 1 mg/Kg prednisone (Panel B). Data points are means +/- S.D. N = 12/group for both groups, except N = 11 for mdx 3 mg/Kg P-188 at 20 and 22 weeks. P < 0.01 for wild type saline vs. all mdx P-188 NF and P < 0.0001 vs. mdx prednisone. Panel A, Not significant for mdx 3 mg/Kg group vs. mdx saline. Panel B. P < 0.05 for the mdx 1 mg/Kg prednisone group vs. mdx saline. (TIF) [file pone.0134832.s010.tif]

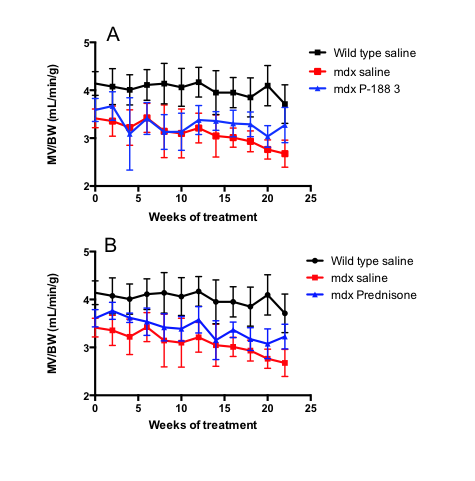

Supplement: S11 Fig — Mdx mice were treated QD, s.c. with P-188 NF or prednisone from age 7 months to 12 months ± 2 weeks. The black line represents wild-type mice (C57BL/10 SnJ) treated with saline. The red line represents mdx mice treated with saline. The blue line represents mice treated with 3 mg/Kg P-188 NF (Panel A) or 1 mg/Kg prednisone (Panel B). Data points are means +/- S.D. N = 12/group for both groups, except N = 11 for mdx 3 mg/Kg P-188 at 20 and 22 weeks. P < 0.0001 for wild type saline vs. all mdx P-188 NF, P < 0.05 for mdx saline vs. mdx P-188 and P < 0.001 for mdx saline vs. mdx prednisone. (TIF) [file pone.0134832.s011.tif]

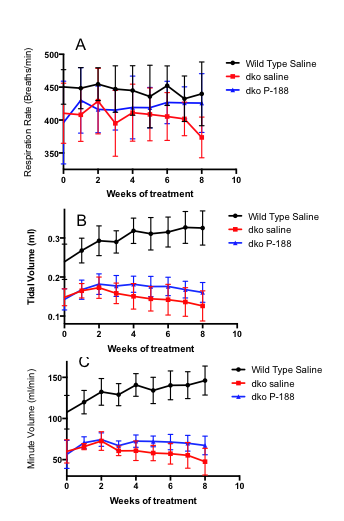

Supplement: S12 Fig — Dko mice were treated with 1 mg/Kg of P-188 NF once per day, s.c, for 8 weeks from ages 3–11 weeks. Respiration was measured weekly by WBP. The key on the right side of each panel identifies the groups and the parameter is indicated on the Y-axis. Data points are means +/- S.D. N = 8/group wild type and 5/group dko saline and dko P-188. P < 0.0001 for wild type saline vs. All mdx groups. Panel A, P < 0.05 for mdx P-188 NF vs. mdx saline. Panel B, P < 0.001 for mdx P-188 NF vs. mdx saline. Panel C, P < 0.0001 001 for mdx P-188 NF vs. mdx saline. N = 18 for wild type saline and N = 9 for mdx P-188 NF. (TIF) [file pone.0134832.s012.tif]

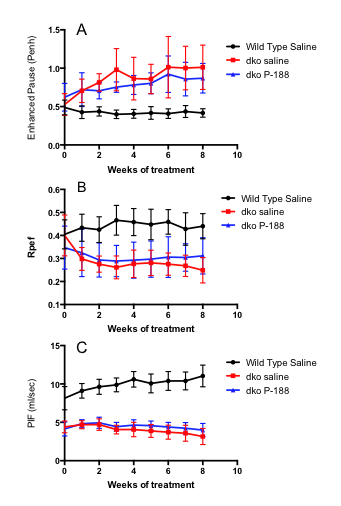

Supplement: S13 Fig — Dko mice were treated with 1 mg/Kg of P-188 NF once per day, s.c, for 8 weeks from ages 3–11 weeks. Respiration was measured weekly by WBP. The key on the right side of each panel identifies the groups and the parameter is indicated on the Y-axis. Data points are means +/- S.D. N = 8/group wild type and 9/group dko saline and dko P-188. P < 0.0001 for wild type saline vs. All mdx groups. The significance for any differences observed can be found in Table 3 in the P-188 (N = 5) row. Panel A, P < 0.01 for mdx P-188 NF vs. mdx saline. Panel B, Not significant for mdx P-188 NF vs. mdx saline. Panel C, P < 0.05 for mdx P-188 NF vs. mdx saline. N = 18 for wild type saline and N = 9 for mdx P-188 NF. (TIF) [file pone.0134832.s013.tif]

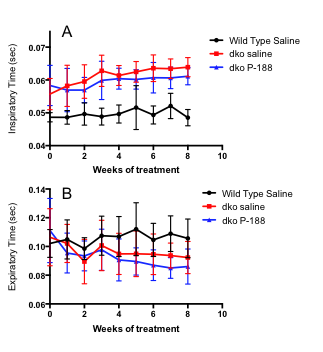

Supplement: S14 Fig — Dko mice were treated with 1 mg/Kg of P-188 NF once per day, s.c, for 8 weeks from ages 3–11 weeks. Respiration was measured weekly by WBP. The key on the right side of each panel identifies the groups and the parameter is indicated on the Y-axis. Data points are means +/- S.D. N = 8/group wild type and 9/group dko saline and dko P-188. P < 0.0001 for wild type saline vs. All mdx groups. The significance for any differences observed can be found in Table 3 in the P-188 (N = 5) row. Panel A, P < 0.05 for mdx P-188 NF vs. mdx saline. Panel B, Not significant for mdx P-188 NF vs. mdx saline. (TIF) [file pone.0134832.s014.tif]

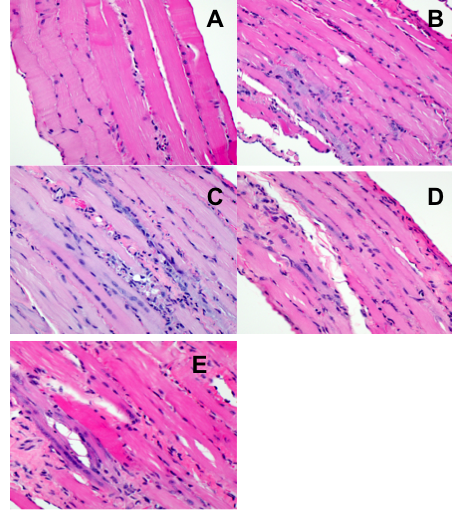

Supplement: S15 Fig — Sections of diaphragms from the same groups of mice described in Fig 1 were stained with H&E. All staining was done on diaphragm muscle from 12-month old mice with the exception of Panel B, which shows a diaphragm from a 7 month old untreated mdx mouse. Shown in the figure are longitudinal sections of diaphragm muscle from: Panel A, wild type control saline-treated mouse; Panel B, a 7 month old mdx mouse; Panel C, an mdx saline-treated mouse; Panel D, an mdx mouse treated with 3 mg/Kg P-188 NF; Panel E, an mdx mouse treated with 1 mg/Kg prednisone. (TIF) [file pone.0134832.s015.tif]
