# Supplementary material for: Chronic Dosing with Membrane Sealant Poloxamer 188 NF Improves Respiratory Dysfunction in Dystrophic Mdx and Mdx/Utrophin-/- Mice
Source: PLoS One. 2015 Aug 6;10(8):e0134832. doi: 10.1371/journal.pone.0134832 (PMC4527695; doi:10.1371/journal.pone.0134832)
Supplement: S1 Table — (DOCX) [file pone.0134832.s016.docx]

S1 Table. Comparison of baseline respiratory function and body weight between groups of wild type and dko mice at 3 weeks of age^g^.

| Parameter | Wild type Saline 1 | dko saline 1 | dko *P-188 1* mg/Kg | dko prednisone |
| --- | --- | --- | --- | --- |
| F (breaths/min) | 450 ± 26^d^ | 410 ± 46^a^ | 396 ± 63^a^ | 413 ± 28^a^ |
| TV (ml) | 0.23 ± 0.04^f^ | 0.15 ± 0.02^c^ | 0.14 ± 0.02^c^ | 0.13 ± 0.02^c^ |
| TV/BW (mL/Kg) | 11.4± 0.77 | 10.5 ± 1.6 | 10.6 ± 1.3 | 9.4± 1.6 |
| MV (ml/min) | 107.6 ± 20^f^ | 60.0 ± 14^c^ | 56.4 ± 17^c^ | 53 ± 8^c^ |
| MV/BW (ml/min/g) | 5.2 ± 0.39^f^ | 4.23 ± 0.65^c^ | 4.11 ± 0.58^c^ | 4.04 ± 0.76^c^ |
| Penh | 0.489 ± 0.096 | 0.527 ± 0.141 | 0.622 ± 0.180^a^ | 0.564 ± 0.132 |
| Rpef | 0.40 ± 0.06 | 0.40 ±0.09 | 0.350 ± 0.09 | 0.385 ± 0.076 |
| PIF (ml/sec) | 8.14 ± 1.52^f^ | 4.41± 0.76^c^ | 4.14 ± 0.91^c^ | 3.79 ± 0.60^c^ |
| PEF (ml/sec) | 4.23 ± 0.97^f^ | 2.36 ± 0.60^c^ | 2.26 ± 0.69^c^ | 2.08 ± 0.26^c^ |
| Ti (sec) | 0.049 ± 0.001^e^ | 0.056 ± 0.005^b^ | 0.058 ± 0.006^c^ | 0.057 ± 0.003^c^ |
| Te (sec) | 0.120 ± 0.010 | 0.106 ± 0.020 | 0.111 ± 0.022 | 0.102 ± 0.011 |
| BW (g) | 20.9 ± 3.0^e^ | 14.5± 4.0^b^ | 13.8 ± 3.9^b^ | 11.3 ± 2.1^c^ |

^a^ P < 0.05 vs. wild type saline

^b^ P = 0.001- 0.01 vs. wild type saline

^c^ P < 0.001 vs wild type saline

^d^ P < 0.05 vs. dko saline

^e^ P = 0.001- 0.01 vs. dko saline

^f^ < 0.001 vs. dko saline

^g^ all values are mean ± S.D. Analysis by one-way ANOVA.
